# Identification of Putative RuBisCo Activase (*TaRca1*)—The Catalytic Chaperone Regulating Carbon Assimilatory Pathway in Wheat (*Triticum aestivum*) under the Heat Stress

**DOI:** 10.3389/fpls.2016.00986

**Published:** 2016-07-12

**Authors:** Ranjeet R. Kumar, Suneha Goswami, Khushboo Singh, Kavita Dubey, Shweta Singh, Renu Sharma, Neeraj Verma, Yugal K. Kala, Gyanendra K. Rai, Monendra Grover, Dwijesh C. Mishra, Bhupinder Singh, Himanshu Pathak, Viswanathan Chinnusamy, Anil Rai, Shelly Praveen

**Affiliations:** ^1^Division of Biochemistry, Indian Agricultural Research InstituteNew Delhi, India; ^2^Division of Genetics, Indian Agricultural Research InstituteNew Delhi, India; ^3^School of Biotechnology, Sher-e-Kashmir University of Agricultural Sciences and TechnologyJammu, India; ^4^Centre for Agricultural Bioinformatics, Indian Council of Agricultural Research-Indian Agricultural Statistics Research InstituteNew Delhi, India; ^5^Nuclear Research Laboratory, Plant Physiology, Indian Agricultural Research InstituteNew Delhi, India; ^6^Center for Environment Science and Climate Resilient Agriculture, Indian Agricultural Research InstituteNew Delhi, India; ^7^Division of Plant Physiology, Indian Council of Agricultural Research-Indian Agricultural Research InstituteNew Delhi, India

**Keywords:** RuBisCo activase, RuBisCo, photosynthesis, heat stress, wheat, IRGA, qRT-PCR, carbon partitioning

## Abstract

RuBisCo activase (Rca) is a catalytic chaperone involved in modulating the activity of RuBisCo (key enzyme of photosynthetic pathway). Here, we identified eight novel transcripts from wheat through data mining predicted to be Rca and cloned a transcript of 1.4 kb from *cv*. HD2985, named as *TaRca1* (GenBank acc. no. KC776912). Single copy number of *TaRca1* was observed in wheat genome. Expression analysis in diverse wheat genotypes (HD2985, Halna, PBW621, and HD2329) showed very high relative expression of *TaRca1* in Halna under control and HS-treated, as compared to other cultivars at different stages of growth. TaRca1 protein was predicted to be chloroplast-localized with numerous potential phosphorylation sites. Northern blot analysis showed maximum accumulation of *TaRca1* transcript in thermotolerant *cv*. during mealy-ripe stage, as compared to thermosusceptible. Decrease in the photosynthetic parameters was observed in all the cultivars, except PBW621 in response to HS. We observed significant increase in the Rca activity in all the cultivars under HS at different stages of growth. HS causes decrease in the RuBisCo activity; maximum reduction was observed during pollination stage in thermosusceptible *cvs*. as validated through immunoblotting. We observed uniform carbon distribution in different tissues of thermotolerant *cvs*., as compared to thermosusceptible. Similarly, tolerance level of leaf was observed maximum in Halna having high Rca activity under HS. A positive correlation was observed between the transcript and activity of *TaRca1* in HS-treated Halna. Similarly, TaRca1 enzyme showed positive correlation with the activity of RuBisCo. There is, however, need to manipulate the thermal stability of TaRca1 enzyme through protein engineering for sustaining the photosynthetic rate under HS—a novel approach toward development of “climate-smart” crop.

## Introduction

Abiotic stresses, such as heat, drought, salinity, radiation, etc. are posing serious threat to agriculturally important crops and food security (Wang D. et al., 2010). Plants employ diverse defense mechanisms to overcome the deleterious effects of the stress and maintain growth and development (Zhu, 2001; Kumar et al., 2013). Heat stress (HS) is one of the major problems affecting the crop productivity (Bita and Gerats, 2013). Heat stress-mediated impairment of photosynthesis is one of the causes for reduction in yield of wheat. It causes an array of morphological, physiological, and biochemical changes such as reduction in the photosynthetic rate, disintegration of photosystem-II, membrane fluidization, denaturation of metabolic and defense related enzymes, drying of stigmatic surface, sterility of pollen, improper fertilization and seed-setting, shriveling of the grains, etc. (Kumar and Rai, 2014). Heat stress induces the expression of stress-associated genes (SAGs) coding for signaling proteins (CDPK, MAPK, etc.), heat shock proteins (low- and high-molecular weight), and antioxidant enzymes (SOD, CAT, POX, etc.) involved in imparting the thermotolerance of the crops (Kotak et al., 2007; Kumar et al., 2012).

Photosynthesis is highly heat-sensitive resulting in the considerable loss of productivity under HS (Wahid et al., 2007). Heat stress inactivates Ribulose-1, 5-bisphosphate carboxylase/oxygenase (RuBisCo), the primary rate limiting enzyme of the carbon assimilatory process (Salvucci and Crafts-Brandner, 2004a).

RuBisCo constitute about 25% of the leaf nitrogen and as much as 50% of soluble leaf protein (Parry et al., 2003). It is the key enzyme of carbon assimilatory pathway and exhibits maximum activity when all its active sites are in proper conformation. RuBisCo enzyme has L8S8 structure comprising eight large (52 kDa) and eight small (14–15 kDa) subunits (Andersson and Backlund, 2008). L8S8 structure of RuBisCo provides greater specificity for RuBP carboxylation than for its oxygenation activity (Jordan and Ogren, 1981). It binds to RuBP-derived enediol acceptor, before binding to the gaseous CO_2_ substrate (Cleland et al., 1998); besides, it has active-site with high affinity for transition-state intermediate, in which the incoming CO_2_ resembles a carboxylate group (Tcherkez et al., 2006). The stability of the RuBisCo enzyme–intermediate complex is very high and it also causes strict binding of phosphate analogs (2-carboxy-D-arabinitol 1-phosphate, xylulose-1, 5-bisphosphate and D-glycero-2, 3-pentodiulose-1, 5-bisphosphate) causing low catalytic turnover of the RuBisCo (Kim and Portis, 2004). The binding of phosphate analogs and RuBP to the active site of RuBisCo is tight enough to inactivate the enzyme by locking the active site in a closed conformation (Salvucci and Crafts-Brandner, 2004b). The rate of deactivation of RuBisCo has been observed to increase with the increase in the temperature (Salvucci and Crafts-Brandner, 2004b). Several studies on cotton, tobacco, Arabidopsis, pea, and wheat have demonstrated that decreased photosynthetic rate during the leaf stress is due to the inactivation of RuBisCo (Law et al., 2001; Salvucci and Crafts-Brandner, 2004c). The closed conformation of the active site of RuBisCo, due to unproductive binding of sugar phosphates, requires conformational remodeling by the RuBisCo activase (Portis, 2003). RuBisCo activase (Rca) is ATPases Associated with diverse cellular Activities (AAA+), which utilizes the energy from ATP to remodel the conformation by releasing the tightly-bound sugar phosphate, and restore its activity. Rca is a nuclear-encoded chloroplastic enzyme discovered by Salvucci et al. (1985) in higher plants. Rca is relatively abundant constituting about 5% of the soluble leaf protein (He et al., 1997). Under the optimal conditions, it removes the inhibiting sugar phosphates that block RuBisCo active sites, and restores RuBisCo to its fully functional state (Portis, 2003). Rca is highly heat-labile (Salvucci et al., 2001). RuBisCo deactivation occurs at temperatures above 30–32°C, in most plants and at higher temperature (>40°C), heat-induced loss of RuBisCo activity becomes irreversible (Kim and Portis, 2004). Some of the researchers reported that Rca under the favorable condition maintains the RuBisCo in its fully activated state, but it does not compensate for the high rate of RuBisCo deactivation during HS (Salvucci and Crafts-Brandner, 2004a), and as a result, the photosynthetic rate decreases, constraining the carbon assimilatory process.

Wheat, being heat-sensitive, is severely affected by HS, especially during flowering and grain-filling stages; HS drastically reduces the growth and yield, by disturbing the source-to-sink ratio and tolerance of the plant (Kumar et al., 2015). Very limited information is, however, available on Rca, its interaction with RuBisCo, and correlation with photosynthetic rate and tolerance level in wheat (*Triticum aestivum*) under HS. The present study aimed at identification of RuBisCo activase gene(s) and elucidation of their role in the carbon assimilatory process of wheat under HS.

## Materials and methods

### Plant material and stress treatment

Seeds of thermotolerant (HD2985 and Halna) and thermosusceptible (PBW621 and HD2329) wheat *cvs*. were procured from Division of Genetics, Indian Agricultural Research Institute (IARI), New Delhi. Seeds pre-treated with Bavistin @ 0.5% were sown in pots (24 pots per cultivar) inside the climate regulated chamber with day/night temperature of 22/18°C, RH of 75% and light intensity of 250 μmol^−2^ s^−1^. Mixture of perlite, FYM and fine sand were used for the pot-filling. Pots were divided into two groups — one set for the control (22 ± 2°C) and another set for the HS treatment (42°C, 2 h). Plants were exposed to HS inside microprocessor regulated chambers in a sinusoidal mode with an increment of 1°C for every 10 min, till it reaches the HS-treated temperature. After HS, the temperature was, thereafter, lowered down to the ambient in the same fashion. Leaf samples were collected, in triplicates, from each stage of development (vegetative, anthesis, milky-ripe, and mealy-ripe) following the Feekes scale (Large, 1954), frozen in liquid nitrogen and stored at –80°C for further downstream analysis. The work plan is presented in the supplementary file (Figure S1).

### RNA-seq for the identification of novel *Rca* transcripts

*De novo* transcriptome analysis of control and HS-treated samples of wheat *cvs*. HD2985 and HD2329 were carried out using Illumina Hiseq 2000 to identify heat-responsive genes (BioProject accession no. PRJNA171754). The unigenes were annotated using different databases and the transcript sequences coding for protein with RuBisCo activase-specific domain were mined from the data; we found 10 different transcripts with AAA+ ATPase conserved domain. All the predicted *Rca* transcripts were aligned using ClustalW alignment tool and we observed that eight of the transcripts have more than 88% similarities among them, whereas two of the transcripts showed very low similarity with others. These transcripts which showed low similarity from others were targeted for the cloning and characterization.

### Molecular cloning of Rca gene

#### Transcript-specific oligo's designing

The transcripts predicted to be *Rca* were used for the primer designing using Prime Perfect Tool (Invitrogen, UK) applying all the default parameters and the primers were subjected to quality check using OligoCalc (http://www.basic.northwestern.edu/biotools/oligocalc.html); HPLC-purified oligo's were synthesized commercially (Table S1).

#### Isolation of total RNA and cDNA synthesis

Total RNA was isolated from the control and HS-treated leaf samples of wheat *cv*. HD2985 by the Trizol method (Invitrogen, UK) and the quality was checked using Bioanalyzer (Agilent, UK); RNA samples with OD 260/280 ratio of more than 2.0 was used for the cDNA synthesis. The integrity of isolated RNA was also checked on 1.2% agarose gel. cDNA was synthesized using the RevertAid™ H minus First Strand cDNA synthesis kit (Thermo fisher Scientific, USA), following the manufacturers protocol and its quality was checked with Qubit™ 2.0 Fluorometer (Invitrogen, UK).

### Cloning and reverse transcriptase-PCR (RT-PCR) analysis of Rca

The transcript-specific oligo's were used for the RT-PCR amplification using the cDNA templates of the control and HS-treated samples with 2x PCR master mix (Promega, Madison, UK). The PCR cycle followed was 98°C for 4 min, followed by 35 cycles of 94°C for 30 s, 58°C for 45 s, and 72°C for 1 min. Further, treatment of 72° for 10 min was given for the stabilization, followed by hold at 4°C. The amplified product was loaded on to 1% agarose gel and an amplicon of ~1.4 kb was observed; the product was eluted from the gel and cleaned with PCR clean-up kit (Promega, Madison, UK); the eluted product was cloned in pGEM-T Easy vector (Promega, Madison, UK) and transformed in *E. coli* strain DH5α competent cells, following the standard protocol (Sambrook et al., 1989). The cloned gene was subjected to restriction analysis, and sequenced using Sanger's di-deoxy method using T7 and SP6 primers.

### *In silico* characterization of the cloned *TaRca1*

The cloned TaRca1 gene was characterized for its homology by using BLASTn tool of National Center for Biotechnology Information (NCBI; https://blast.ncbi.nlm.nih.gov/). The nucleotide sequence was submitted to GenBank (http://www.ncbi.nlm.nih.gov/genbank/). Translated sequence of the cloned TaRca1 gene was predicted using Expasy tool (http://expasy.org/tools/); open reading frame (ORF) was predicted using ORF Finder (http://www.ncbi.nlm.nih.gov/projects/gorf/). The conserved domain (CD) of the gene was searched with CD-search tool of NCBI (http://www.ncbi.nlm.nih.gov/Structure/cdd/wrpsb.cgi).

### Southern blot analysis to know the copy number of cloned TaRca1 gene

Southern blot analysis was carried out in thermotolerant wheat *cv*. HD2985. Genomic DNA was isolated from the etiolated seedlings of *cv*. HD2985 by cetyl-trimethylammonium bromide (CTAB) method (Procunier et al., 1990). Twelve microgram of genomic DNA was digested with the restriction endonucleases *Eco*RI, *Hin*DIII, and *Bam*HI (HF restriction enzyme, NEB, UK) overnight at 37°C and resolved on 0.8% agarose gel (15 V for 12 h). The resolved DNA was blotted onto a piece of nylon membrane (Hybond-N^+^, Amersham Biosciences, Uppsala, Sweden) using iBlot™ (Invitrogen, UK). DNA on the blot was denatured with an alkaline buffer (0.5 N NaOH, 1.5 M NaCl) for 2 min, soaked in a neutralizing buffer (0.5 M Tris HCl [pH 7.5], 1.5 M NaCl) for 2 min the membrane was baked at 80°C for 1 h. The baked membrane was pre-hybridized in a pre-hybridization buffer [0.5% sodium dodecyl sulfate (SDS), 6X SSC, 5X denhardt's solution] containing 100 μg of salmon sperm DNA (Pharmacia, Uppsala, Sweden) per ml at 65°C for 4 h. The probe (*TaRca1* fragment of ~600 bp generated after the restriction of the gene) was labeled with biotin labeling mix (Fermentas, UK) and was used for the hybridization of the membrane at 65°C for 15 h inside the Hybridizer. The hybridized membrane was washed twice with 0.1% SDS in 2 × SSC (150 mmol/L NaCl, 15 mmol/L sodium citrate) solution for 10 min at room temperature, and then twice with 0.1% SDS in 0.2 × SSC solution for 20 min at 65°C. Biotin-labeled probe-target hybrids were detected with alkaline phosphatase-conjugated streptavidin using the Biotin Chromogenic Detection kit (Thermo fisher Scientific, USA). The blot developed was immediately washed in water and the image was captured using Gel Doc Easy (Bio Rad, UK).

### Northern blot analysis

Expression analysis of cloned *TaRca1* gene was carried out in contrasting wheat *cvs*. HD2985 (thermotolerant) and H2329 (thermosusceptible) by using northern blotting. Total RNA was isolated from the collected samples by the Trizol method (Invitrogen, UK) and quantified with Qubit™ 2.0 Fluorometer (Invitrogen, UK); the integrity was verified on 1.2% agarose gel. For northern blot analysis, 6 μg of total RNA was loaded onto 1.2% formaldehyde agarose gel, and run at 45 V for 2 h. The resolved RNA was blotted on to Nylon membrane using iBlotter (Invitrogen, UK). The membrane was UV-cross linked and baked at 80°C for 1 h. Further, the membrane was pre-hybridized in a hybridization buffer (1% SDS, 1.5 M NaCl, 10% dextran sulfate) containing 100 μg of salmon sperm DNA (Pharmacia, Uppsala, Sweden) per mL at 65°C for 4 h. RuBisCo activase (*TaRca1*) DNA was labeled with α-[^32^P]-dCTP (BRIT, Bhabha Atomic Research Centre, India) and was used as a probe for the hybridization at 65°C for 15 h. The membrane was washed twice with 0.1% SDS in 2 × SSC (150 mmol/L NaCl, 15 mmol/L sodium citrate) solution for 15 min, and then twice with 0.1% SDS in 0.2 × SSC solution for 15 min at 50°C. An X-ray film was exposed to the air-dried radio-labeled membrane, and the signals were quantified using automatic x-ray developer.

### Expression analysis of *Tarca1* using quantitative real-time PCR (qRT-PCR)

Total RNA was isolated from the control and HS-treated samples of wheat *cvs*. HD2985, HD2329, Halna, and PBW621 by the Trizol method (Invitrogen, UK) and was quantified using Qubit™ 2.0 Fluorometer (Invitrogen, UK). First strand cDNA synthesis was performed, using oligo dT primers and the Superscript II reverse transcriptase (Invitrogen, UK) according to the manufacturer's instructions. First-strand cDNA was diluted to a final concentration of 100 ng/μL and real-time PCR was carried out as described earlier (Kumar et al., 2013). Expression analysis was carried out in three biological and three technical replicates. The expression levels of wheat β-actin gene (accession no. AF282624) was used for normalizing the *Ct*-value. The comparative C_t_ (2^−ΔΔCt^) method was used to calculate the relative expression of the *TaRca1* (Pfaffl et al., 2002).

### Photosynthetic rate and stomatal conductance analysis

The wheat *cvs*. HD2985, HD2329, PBW621, and Halna under control (22 ± 3°C) and HS (38°C, 2 h) during the grain-filling (Feekes—11.1) stage were subjected to infra-red gas analyzer (IRGA) for analyzing the effect of HS on the photosynthesis (LiCor 6400, LiCor Inc., USA), and the observations were recorded following Long and Bernacchi (2003). Fully expanded flag leaf was used for the IRGA analysis, and the readings were taken in triplicates between 11 a.m. and 12 p.m. Photosynthesis was measured at constant saturating light of 1500 μmol m^−2^ s^−1^. Measurements of leaf R_dark_ were made on dark-adapted leaves after 30 min of dark adaptation to achieve steady-state R_dark_.

### RuBisCo activity assay

The leaf samples collected from the wheat *cvs*. HD2985, HD2329, PBW621, and Halna (control and HS-treated) during anthesis, milky-ripe, and mealy-ripe stages were used for the activity assay. One gram leaf was crushed into fine powder using liquid nitrogen in 5 mL extraction buffer [50 mM Tris-HCl (pH 8.0), 1 mM EDTA, 1 mM PMSF, 0.1% BSA]. The extract was centrifuged at 9000 g for 15 min and the supernatant was decanted into separate tubes. One milliliter supernatant was activated by incubating at RT in the activation buffer [0.1 mL each of 10 mM NaHCO_3_, 10 mM MgCl_2_, 5 mM glutathione, 0.1 mM EDTA, and 0.6 mL Tris-HCl (50 mM) pH 8.0]. Reaction mixture was prepared by adding 0.2 mL Tris-HCl (pH 8.0), 0.1 mL labeled NaH^14^CO_3_ (20 mM, specific activity 50 mCi mmol^−1^, BARC, Mumbai), and 0.1 mL unlabeled NaHCO_3_,0.01 ml MgCl_2_ (3.03 μmole), 0.01 ml EDTA, 0.1 mL activated enzyme extract, and 0.1 mL RuBP (0.2 μmole). The reaction was started by addition of RuBP and terminated after 120 min with addition of 0.2 mL 6N acetic acid. The contents were evaporated to dryness at 65°C and the acid stable ^14^C was counted using liquid scintillation counter (Packard, Tri-Carb, 1600 TR USA). Assay medium without RuBP served as blank for each assay.

### Immunoblot assay of RuBisCo

The control and HS-treated leaf samples collected from wheat *cvs*. HD2985 and HD2329 during the vegetative, pollination, and grain-filling were subjected to western blot analysis described earlier (Kumar et al., 2013). The dilution used for the incubation of membrane in primary monoclonal antibody (anti-RuBisCo) and secondary antibody (peroxidase-conjugated goat anti-mouse IgG) was 1:1000. Band of desired intensity was visible after 2–5 min incubation, and the membrane was washed immediately in water, and Tris-buffered saline (TBS). The dried membrane was used for photography with Gel Doc Easy (Bio Rad, UK).

### RuBisCo activase activity assay

Rca activity was estimated in control and HS-treated leaf samples of wheat *cvs*. HD2985, HD2329, PBW621, and Halna collected at pollination, milky-ripe, and mealy-ripe stages. Fresh leaf (0.3 g) was crushed in 600 μL of extraction buffer [50 mM Tricine buffer (pH-7), 100 mM MgCl_2_, 10 mM NaHCO_3_, 1 mM EDTA, 1 mM ATP, 10 mM DTT, 1 mM PMSF, 2 mM Benzamide, 0.01 mM Leupeptin] and centrifuged at 13,000 rpm for 20 min. The supernatant collected was used for the protein estimation by Bradford method (Bradford, 1976). For the activity assay of Rca, 40 μg of extract was taken along with denatured crude extract (incubated at 65°C for 10 min) and 25 μL of assay medium [100 mM Tricine buffer (pH-8), 10 mM MgCl_2_, 10 mM NaHCO_3_, 1 mM ATP, 4 mM RuBP, 1 mM Phosphoenolpyruvate, Pyruvate kinase (20 U/ml)] and 25 μL of RuBP-RuBisCo complex (1 mg RuBP and 1 mg RuBisCo was taken and dissolve in 1.5 mL of water, and kept at 40°C for 30 min) was added in both the scintillation vials. The reaction was allowed for 30 s, and further, 450 μL of radio-labeled assay mix [100 mM Tricine buffer (pH-8), 10 mM MgCl_2_, 0.5 mM RuBP, 20 mM NaH^14^CO_3_(specific activity 50 mCi mmol^−1^, BARC, Mumbai)] was added. The reaction was terminated by adding 100 μL of 1N HCl. The contents of the vials were evaporated to dryness at 65°C and the acid stable ^14^C was subsequently counted in the liquid scintillation counter (Packard, Tri-Carb, 1600 TR USA). The RCA activity was calculated by comparing the disintegration per minute (DPM) of samples (control—denatured) and NaH^14^CO_3_.

### Estimation of reducing, non-reducing, and total sugars

The samples (control and HS-treated) collected from wheat *cvs*. HD2985, HD2329, PBW621, and Halna during grain-filling (milky-ripe and mealy-ripe) stage were used for the estimation of reducing, non-reducing, and total sugars. Reducing sugars from the fresh leaves and stems were estimated by the 3, 5-dinitrosalicylic acid (DNS) method (Miller, 1959). Fresh samples (0.1 g) were homogenized in 80% ethanol and centrifuged at 5000 g for 15 min at the room temperature; the supernatant was used for the sugars estimation using the standard of glucose. Non-reducing sugar was estimated by acid hydrolysis of the samples in sulfuric acid. Total sugar was estimated by phenol sulfuric acid method (Dubois et al., 1956).

### Estimation of total antioxidant capacity (TAC)

Leaves of control and HS-treated samples collected from all the four cultivars at pollination, milky-ripe, and mealy-ripe stages were used for the TAC estimation. TAC was assayed in the fresh leaves as described by Benzie and Strain (1999). The antioxidant capacity was expressed as ferric-reducing ability of 1 mmol/L FeSO_4_.

## Results

### Identification of transcripts predicted to be RuBisCo activase (Rca)

The data generated from the *de novo* whole-transcriptome sequencing of wheat *cvs*. HD2985 and HD2329 (BioProject accession no. PRJNA171754) were mined for the prediction of transcripts coding for Rca. Mining of the RNA-seq data revealed the presence of eight putative *Rca* transcripts based on the domain search (presence of Rca-specific ATPases Associated with diverse cellular Activities superfamily of ring-shaped P-loop NTPases domain; Table S2). The transcript sequence of identified putative Rca genes is presented in the supplementary file (Table S3). Based on the *in silico* characterization and size of the transcripts, we targeted transcript_5 for the cloning and characterization.

### Cloning and *In silico* characterization of putative *Rca*

An amplicon of ~1.4 kb was amplified from wheat *cv*. HD2985 using transcript specific primers. The amplified product was purified and cloned in pGEM-T Easy vector. Sanger's sequencing using di-deoxy method showed the presence of 1402 nucleotides with an open reading frame of 432 aa starting from 23 to 1321 nt. Based on the homology search, the gene was named as *TaRca1*. The nucleotide sequence was submitted in NCBI (GenBank accession no. KC776912).

BLASTn search showed maximum homology with *RcaB* reported from *Triticum* (acc. no. AF251264.1) and cDNA clone (FLbaf169e16) from *Hordeum vulgare* (acc. no. AK252703.1). Protein-based homology search, using BLASTp suite of NCBI, showed maximum (99%) homology with Rca protein reported from *Triticum urartu* (acc. no. EMS57011.1) followed by 93% with *RcaB* reported from *Aegilops tauschii* (acc. no. EMT06779.1).

TaRca1 protein was predicted to be chloroplast-localized. We observed numerous potential phosphorylation sites with serine, threonine, and tyrosine residues in the TaRca1 protein; four sites with tyrosine and two sites with serine and threonine were predicted to have phosphorylating potential above the threshold. Based on the phosphorylating sites, the maximum kinase activity was predicted for the Protein kinase C (PKC)—a family of serine/threonine protein kinase enzymes. A prominent domain was observed between the region 121—354 aa predicted to belong to superfamily P-loop containing nucleoside triphosphate hydrolases and family AAA-ATPase domain.

**Figure 1:**
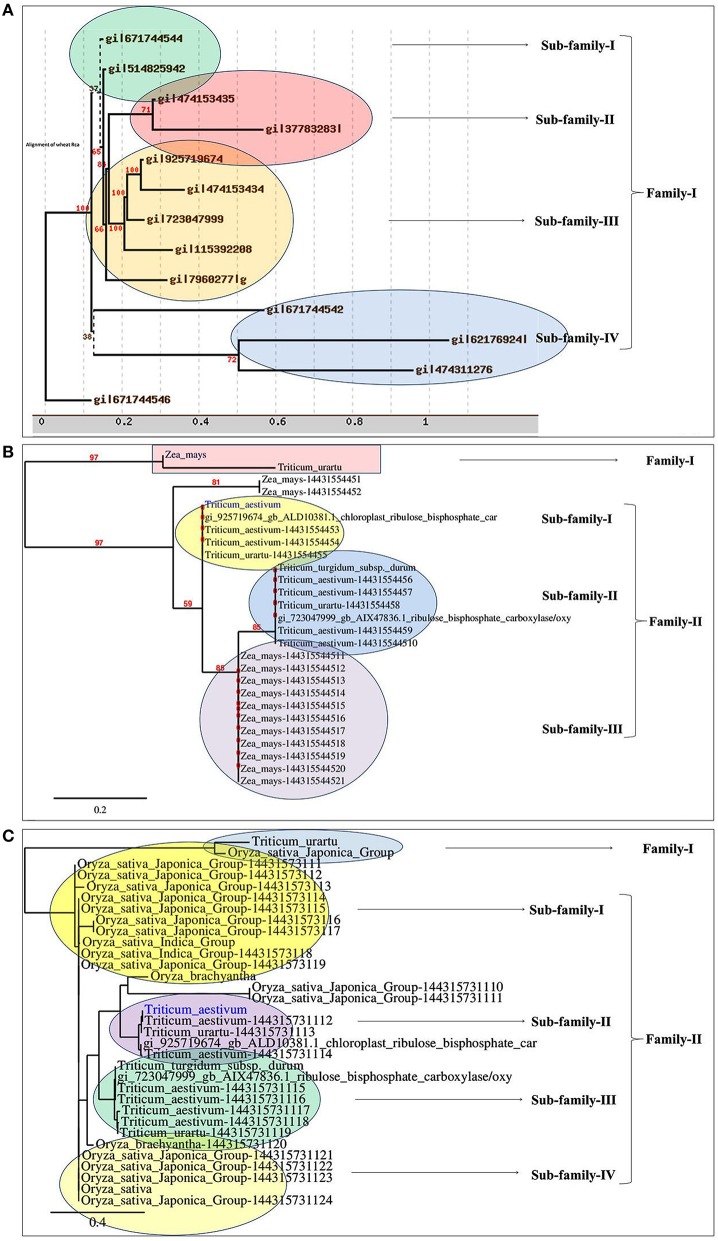
**Phylogenetic analysis of RuBisCo activase gene (***TaRca1***) cloned from wheat ***cv***. HD2985**. **(A)** Classification of *TaRca1* based on RuBisCo activase reported from other plant and non-plant sources, **(B)** Classification based on *Rca* reported from *Zea mays*; **(C)** Classification based on *Rca* reported from *Oryza sativa*.

Based on the phylogeny tree, the *Rca* was classified into four different sub-families with *TaRca1* lying under subfamily—I (Figure 1A). The cloned *TaRca1* was subjected to phylogeny analysis along with RuBisCo activase reported from *Zea mays* (Figure 1B). Based on the amino acid sequence analysis, the RuBisCo activase was broadly classified into two families—(a) Family-I and (b) Family-II, with family-II having three subfamilies; cloned *TaRca1* was observed in subfamily-II of family-I. Similarly, Rca reported from *Oryza sativa* was used for the phylogeny analysis along with Rca reported from wheat (Figure 1C). Rca was classified into two families with family-II having four different subfamilies; cloned *TaRca1* was observed in subfamily-II.

### Southern and northern blot analysis of TaRca1 gene

**Figure 2:**
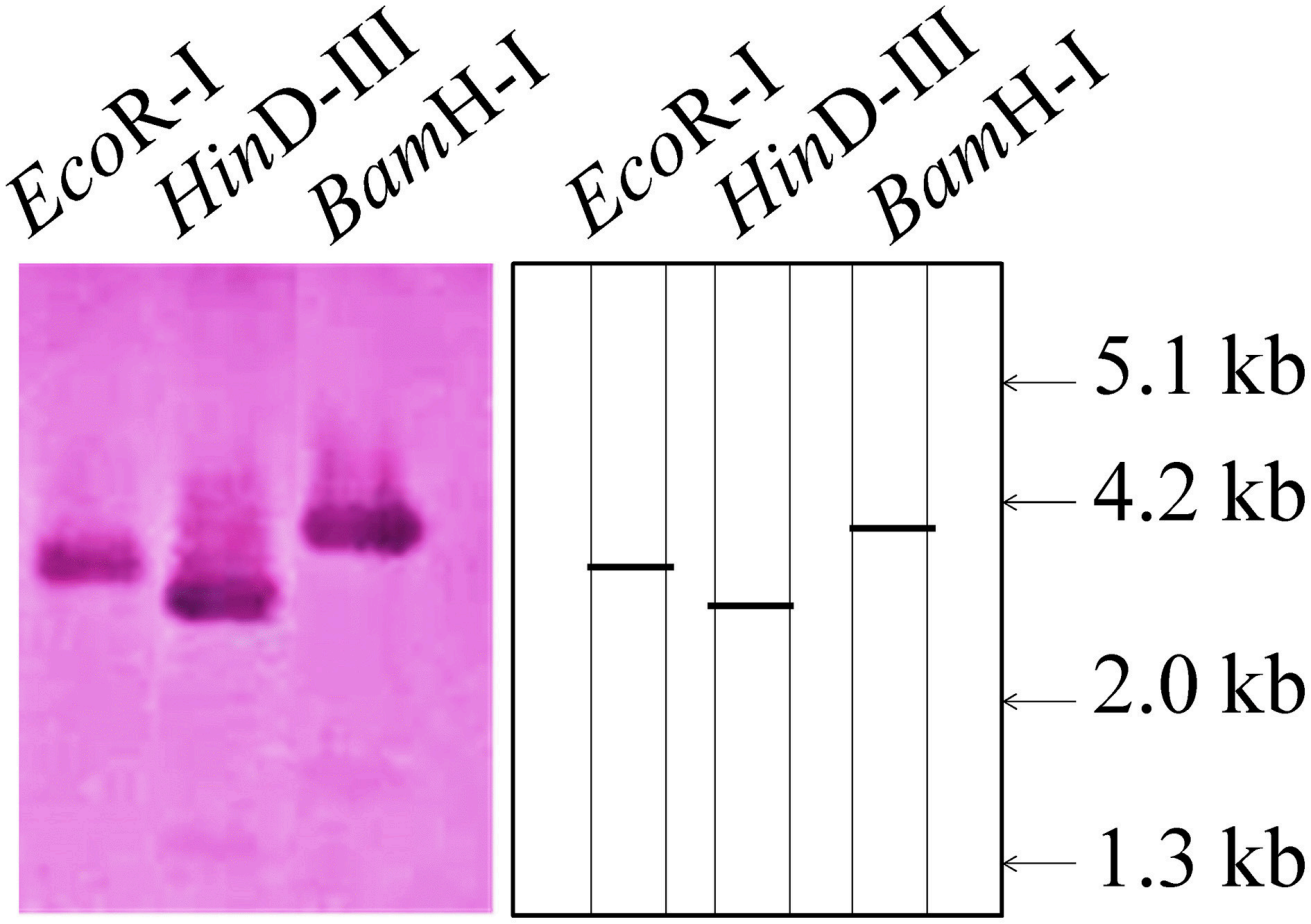
**Southern blot analysis to identify the copy number of cloned RuBisCo activase (***TaRca1***) gene from wheat ***cv***. HD2985; ***Eco***RI, ***Hin***DIII, and ***Bam***HI enzymes were used for the restriction; 12 μg genomic DNA was used per sample; 0.8% agarose gel was used for the electrophoretic separation of restricted product; Biotin-labeled probe was used for the hybridization**.

To find out the copy number of the cloned TaRca1 gene from wheat, southern blot analysis was carried out using the genomic DNA isolated from wheat *cv*. HD2985. We observed single prominent blot in each lane (restricted with *Eco*RI, *Hin*DIII, and *Bam*HI; Figure 2). *Eco*RI restricted lane showed band of ~3.2 kb, whereas *Bam*HI and *Hin*DIII showed bands of ~2.8 and ~4.0 kb. This result suggests that cloned *TaRca1* has single copy in *Triticum aestivum*.

**Figure 3:**
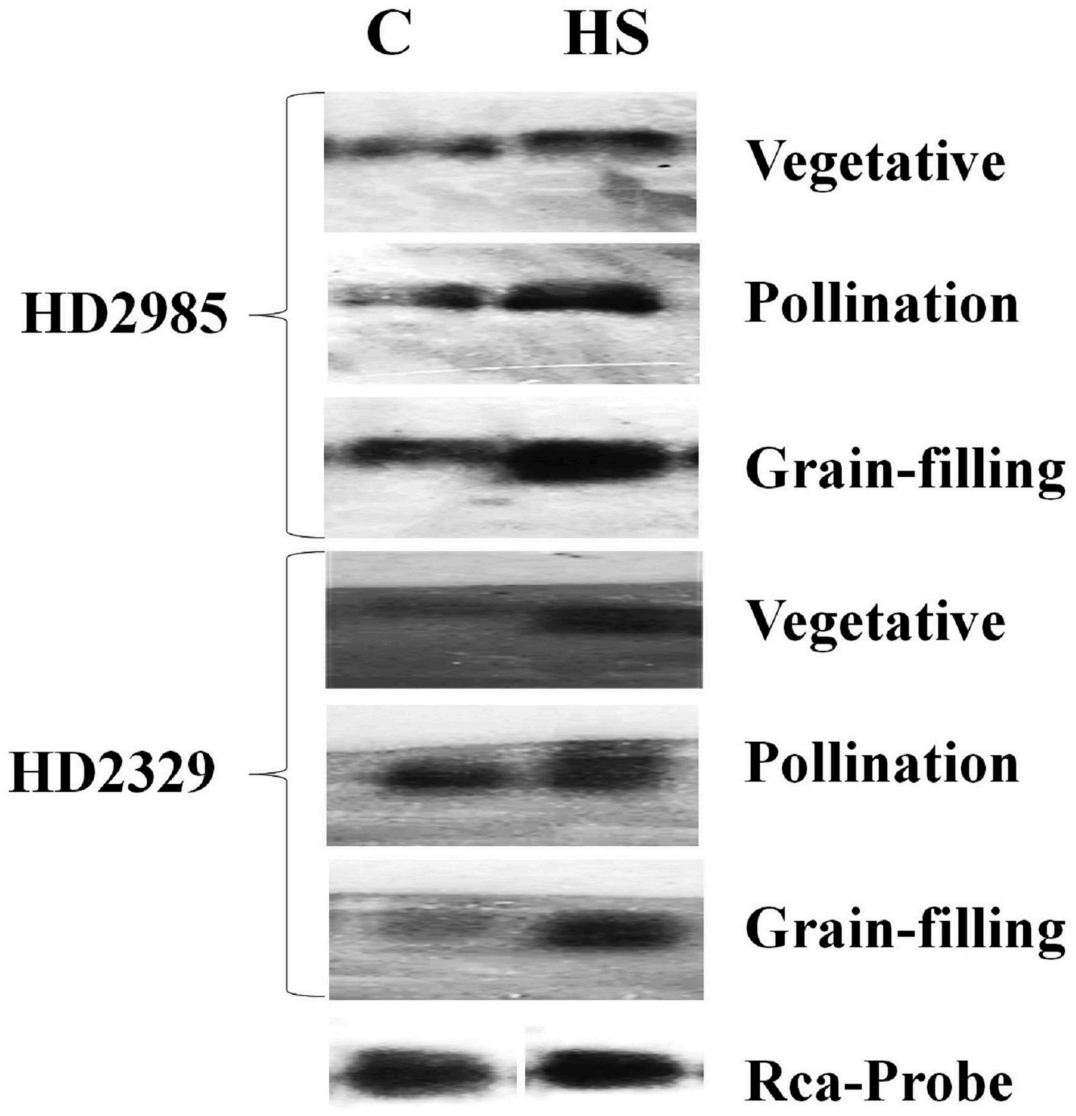
**Northern blot analysis of cloned RuBisCo activase (***TaRca1***) gene in contrasting wheat ***cvs***. HD2985 and HD2329 at different stages of growth and development; [α-^**32**^P]-dCTP (300 μCi) were used for the probe labeling; 1.2% agarose gel was used for the electrophoretic separation of total RNA**.

The expression of cloned *TaRca1* was further analyzed though northern blotting in the contrasting wheat *cvs*. HD2985 and HD2329 under control and HS-treated conditions. We observed significant increase in the expression of *TaRca1* transcripts in HD2985 under HS, as compared with the control at all the stages studied; most prominent accumulation of transcript was observed during grain-filling (mealy-ripe) stage under HS (Figure 3). Similar pattern of expression was observed in *cv*. HD2329; the expression levels of *TaRca1* under HS was, however, lower in HD2329 (thermosensitive) than in HD2985 (thermotolerant).

### Alterations in the photosynthesis-associated parameters under HS

**Figure 4:**
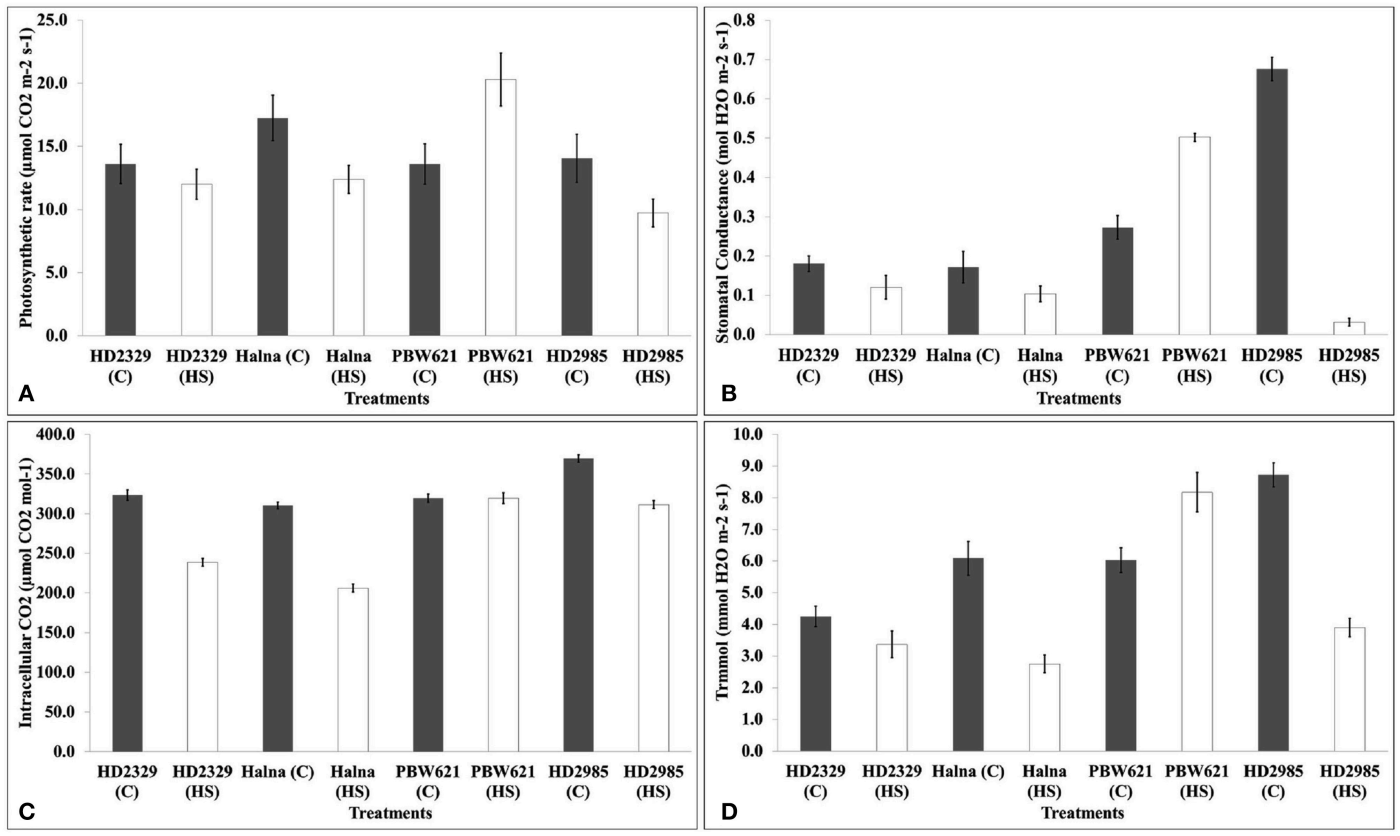
**Infra-Red Gas Analyzer (IRGA) analysis of full expanded leaves of different wheat ***cvs***. under control and heat stress conditions**. **(A)** Photosynthetic rate, **(B)** Stomatal conductance, **(C)** Intracellular carbon dioxide, **(D)** Transpiration rate; C −22 ± 3°, heat stress –42°C for 2 h; Leaves of HD2985, HD2329, Halna and PBW621 were used for the analysis; vertical bars indicate SE (*n* = 3).

We observed significant decrease in the photosynthetic rate in response to HS in HD2985, HD2329, and Halna except PBW621. Decrease in the photosynthetic rate was maximum in Halna and HD2985 (Figure 4). Stomatal conductance also decreased due to HS, in all the cultivars except PBW621; decrease was maximum in HD2985. Significant decrease in the intracellular CO_2_ was observed in response to HS in all the cultivars; differences were, however non-significant in PBW621. Similarly, transpiration rate also decreased due to HS in all the cultivars except PBW621, the decrease being highest in HD2985.

### Expression pattern of *TaRca1* in different wheat genotypes under heat stress

**Figure 5:**
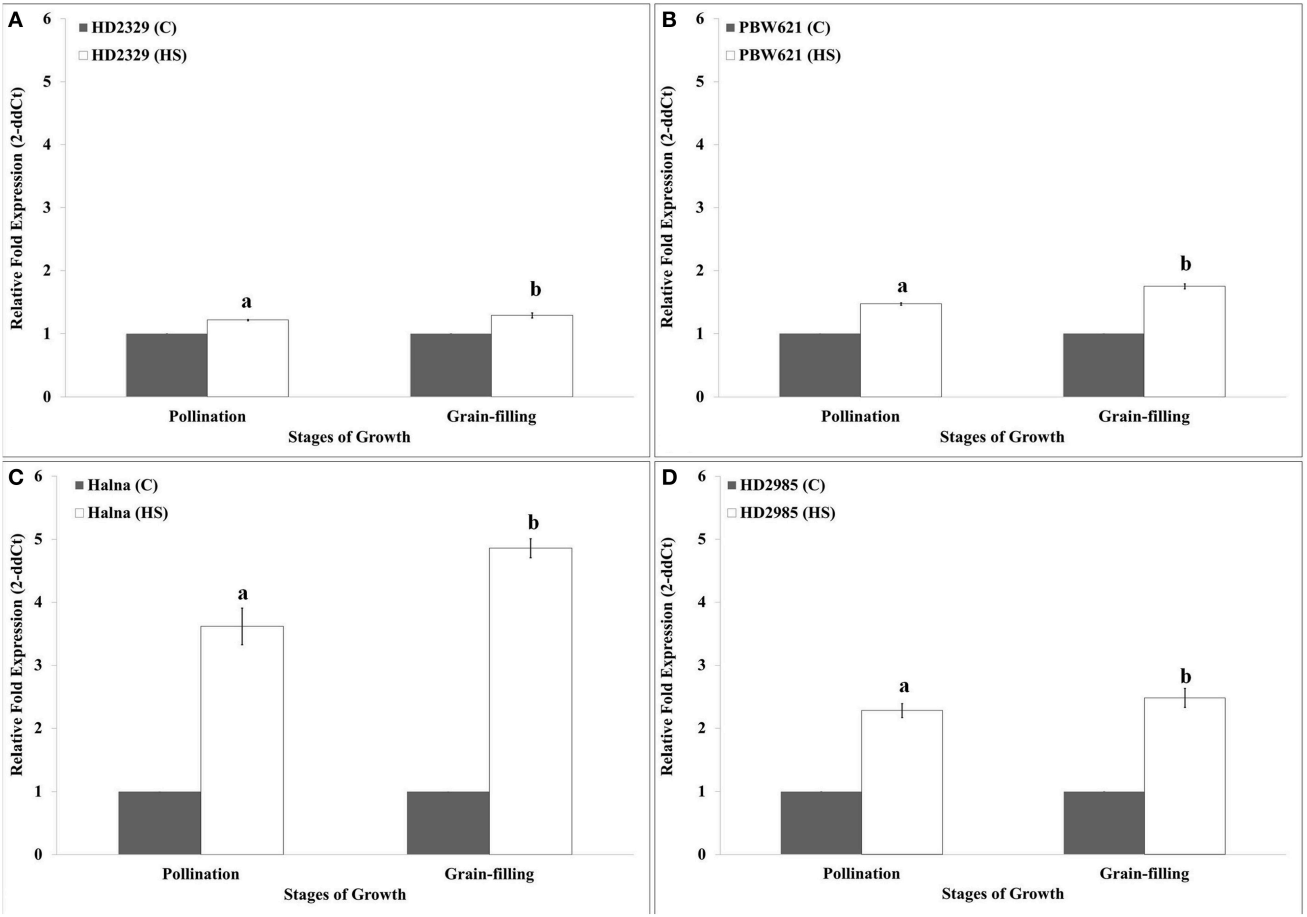
**Expression profiling of RuBisCo activase (***TaRca1***) in wheat during pollination and grain-filling stages under control (C) and heat stress (HS) conditions**. **(A)** HD2329 (C and HS), **(B)** PBW621(C and HS), **(C)** Halna (C and HS), **(D)** HD2985 (C and HS); C −22 ± 3°, heat stress −42°C for 2 h; β-actin gene (accession no. AF282624) was used as endogenous control for normalizing the C_t_ value; Relative expression was calculated by Pfaffl method (Pfaffl et al., 2002); vertical bars indicate SE (*n* = 3).

HD2329 showed very high relative expression of *TaRca1* in response to HS, as compared to control during pollination and grain-filling stages; maximum expression was observed during grain-filling in response to HS (Figure 5A). Similar pattern of expression of *TaRca1* was observed in PBW621, HD2985, and Halna under control and HS-treated conditions (Figures 5B–D). Of all the *cvs*., relative expression of *TaRca1* under HS was observed maximum in Halna and minimum in HD2329 during both the stages of growth. In general, abundance of *TaRca1* transcript was observed during the grain-filling in all the four cultivars in response to HS.

### Changes in the Rca activity under heat stress

**Figure 6:**
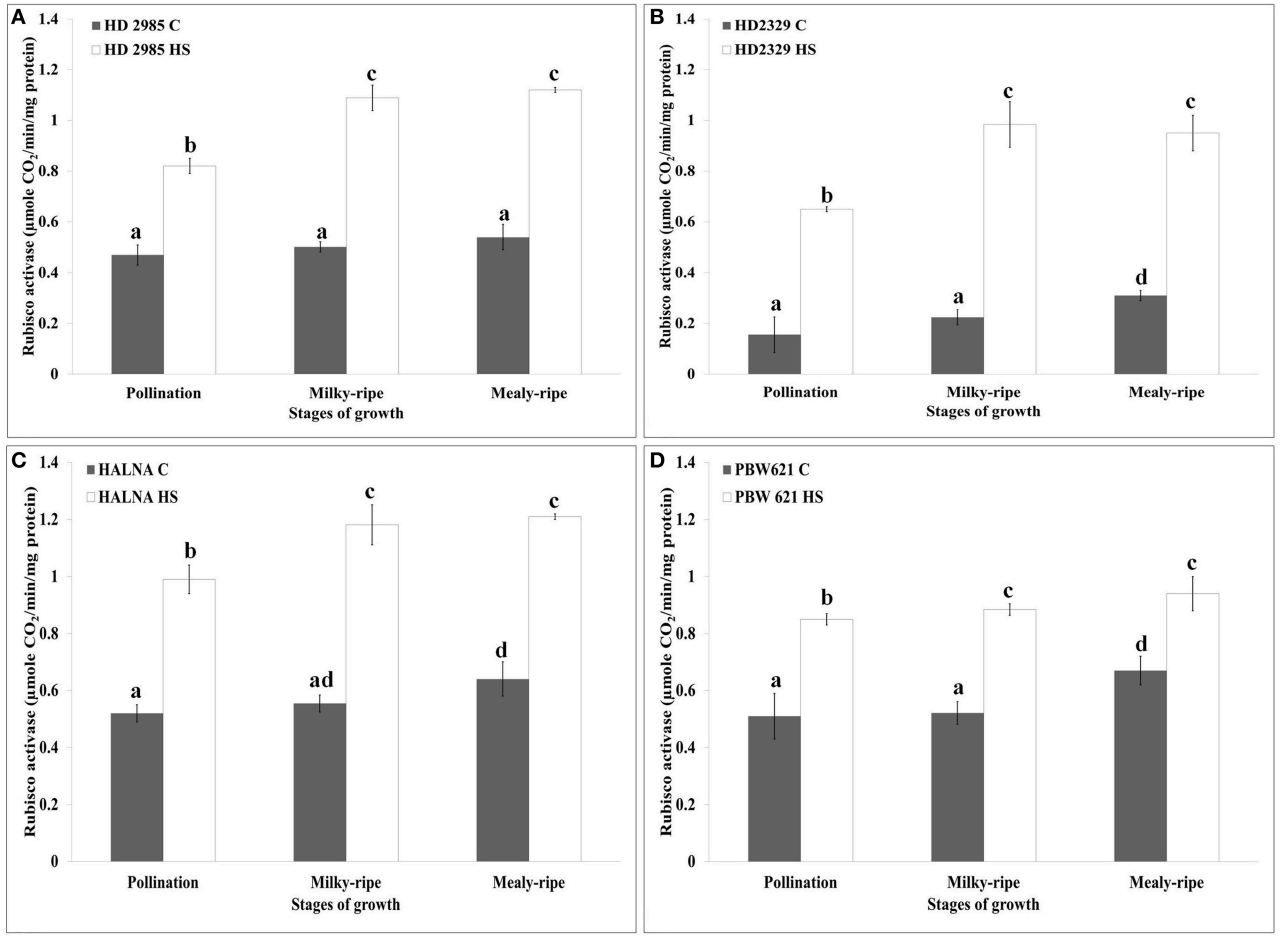
**RuBisCo activase (Rca) activity assay in contrasting wheat cultivars at different stages of growth and development under control (C) and heat stress (HS) conditions. (A)** HD2985 (C and HS), **(B)** HD2329 (C and HS), **(C)** Halna (C and HS), **(D)** PBW621 (C and HS); samples collected from pollination, milky-ripe and mealy-ripe stages were used for the activity assay; C −22 ± 3°, heat stress −42°C for 2 h; NaH^14^CO_3_ (20 mM, specific activity 50 mCi mmol^−1^, BARC, Mumbai) was used for the labeling; different letters above each bar indicate a significant difference (*p* < 0.05) between the treatments (one-way ANOVA); vertical bars indicate SE (*n* = 3).

HD2985 showed maximum Rca activity during mealy-ripe stage, both under control (0.54 μmole CO_2_/min/mg protein) and HS-treated (1.19 μmole CO_2_/min/mg protein) conditions (Figure 6A). We observed significant increase in the Rca activity in HD2985 under HS at different stages of development.

In case of HD2329, we observed maximum Rca activity during milky-ripe stage in response to HS, and mealy-ripe stage under control condition (Figure 6B). Non-significant difference in the Rca activity in HD2329 under HS was observed during milky- and mealy-ripe stages. Similar pattern of Rca activity was observed in Halna under control and HS-treated conditions at all the stages of development. Halna showed maximum Rca activity during mealy-ripe stage under control (0.62 μmole CO_2_/min/mg protein) and HS-treated (1.22 μmole CO_2_/min/mg protein) conditions (Figure 6C). PBW621 showed non-significant difference in the Rca, as observed in HS-treated samples collected during different stages of development. Maximum Rca activity was observed during mealy-ripe stage, both under control (0.69 μmole CO_2_/min/mg protein) and HS-treated (0.94 μmole CO_2_/min/mg protein) conditions (Figure 6D).

### Changes in the RuBisCo activity under heat stress

**Figure 7:**
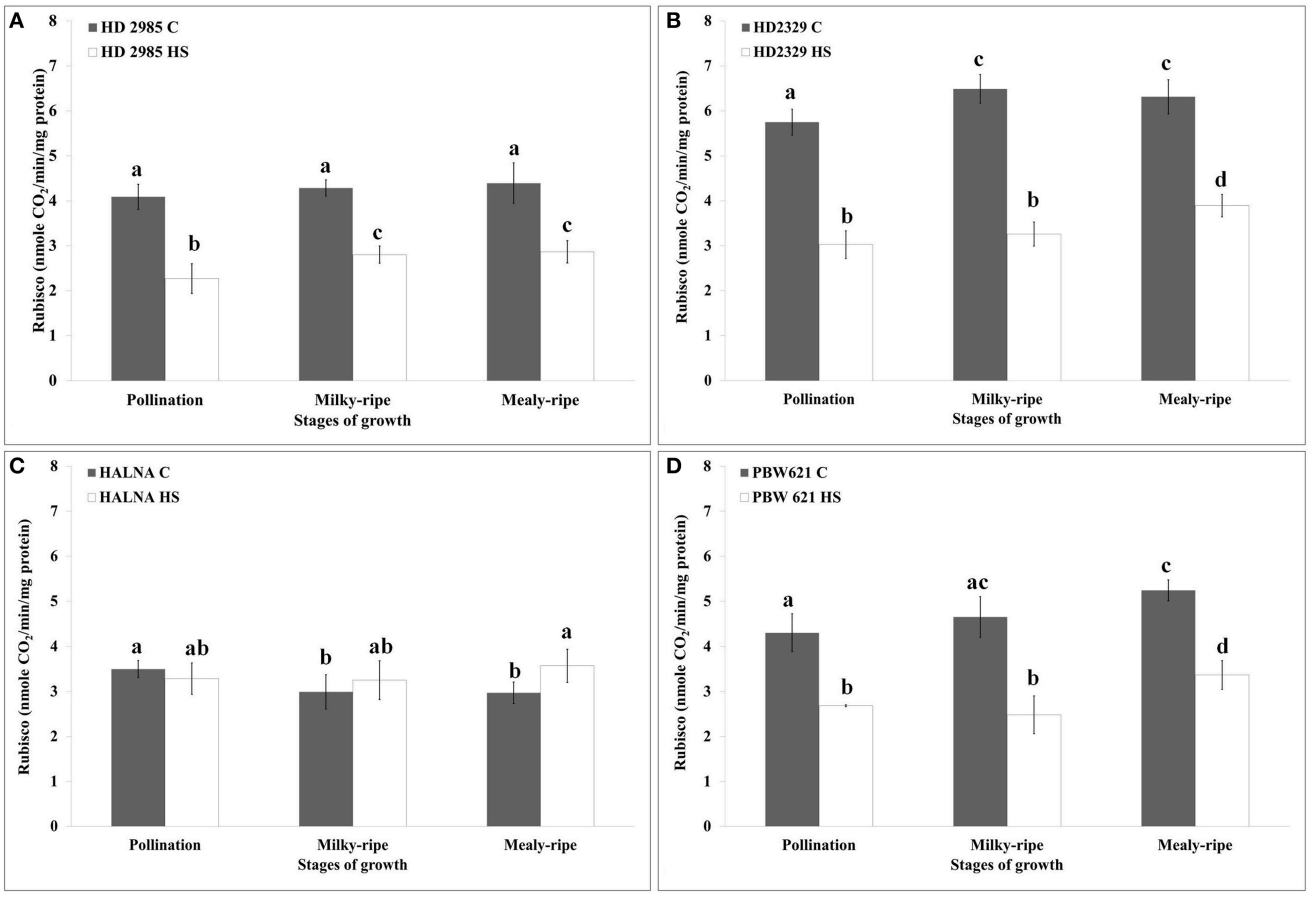
**Variations in the RuBisCo activity in contrasting wheat cultivars under control (C) and heat stress (HS) conditions at different stages of growth. (A)** HD2985 (C and HS), **(B)** HD2329 (C and HS), **(C)** Halna (C and HS), **(D)** PBW621 (C and HS); samples collected from pollination, milky-ripe and mealy-ripe stages were used for the activity assay; C −22 ± 3°, heat stress −42°C for 2 h; NaH^14^CO_3_ (20 mM, specific activity 50 mCi mmol^−1^, BARC, Mumbai) was used for the labeling; different letters above each bar indicate a significant difference (*p* < 0.05) between the treatments (one-way ANOVA); vertical bars indicate SE (*n* = 3).

HD2985 showed maximum RuBisCo activity during mealy-ripe stage, both under control (4.3 nmole CO_2_/min/mg protein) and HS-treated (2.69 nmole CO_2_/min/mg protein) conditions (Figure 7A). HS causes decrease in the RuBisCo activity; maximum reduction was observed during pollination stage. In case of HD2329, we observed maximum RuBisCo activity during milky-ripe stage (6.5 nmole CO_2_/min/mg protein) under control condition and mealy-ripe stage (4 nmole CO_2_/min/mg protein) under HS-treated condition (Figure 7B). Percent reduction in RuBisCo activity in HD2329 under HS was observed maximum during milky-ripe stage, as compared to other stages of development. Halna showed non-significant differences in the RuBisCo activity estimated in control and HS-treated samples at different stages of development (Figure 7C). PBW621 showed similar pattern of RuBisCo activity as shown by HD2985 under control and HS-treated conditions. Maximum RuBisCo activity was observed during mealy-ripe stage, both under control (5.3 nmole CO_2_/min/mg protein) and HS-treated (3.5 nmole CO_2_/min/mg protein) conditions (Figure 7D). Overall, RuBisCO activity was observed maximum in HD2329, both under control and HS-treated conditions at different stages of growth.

### Accumulation pattern of RuBisCo protein under HS

**Figure 8:**
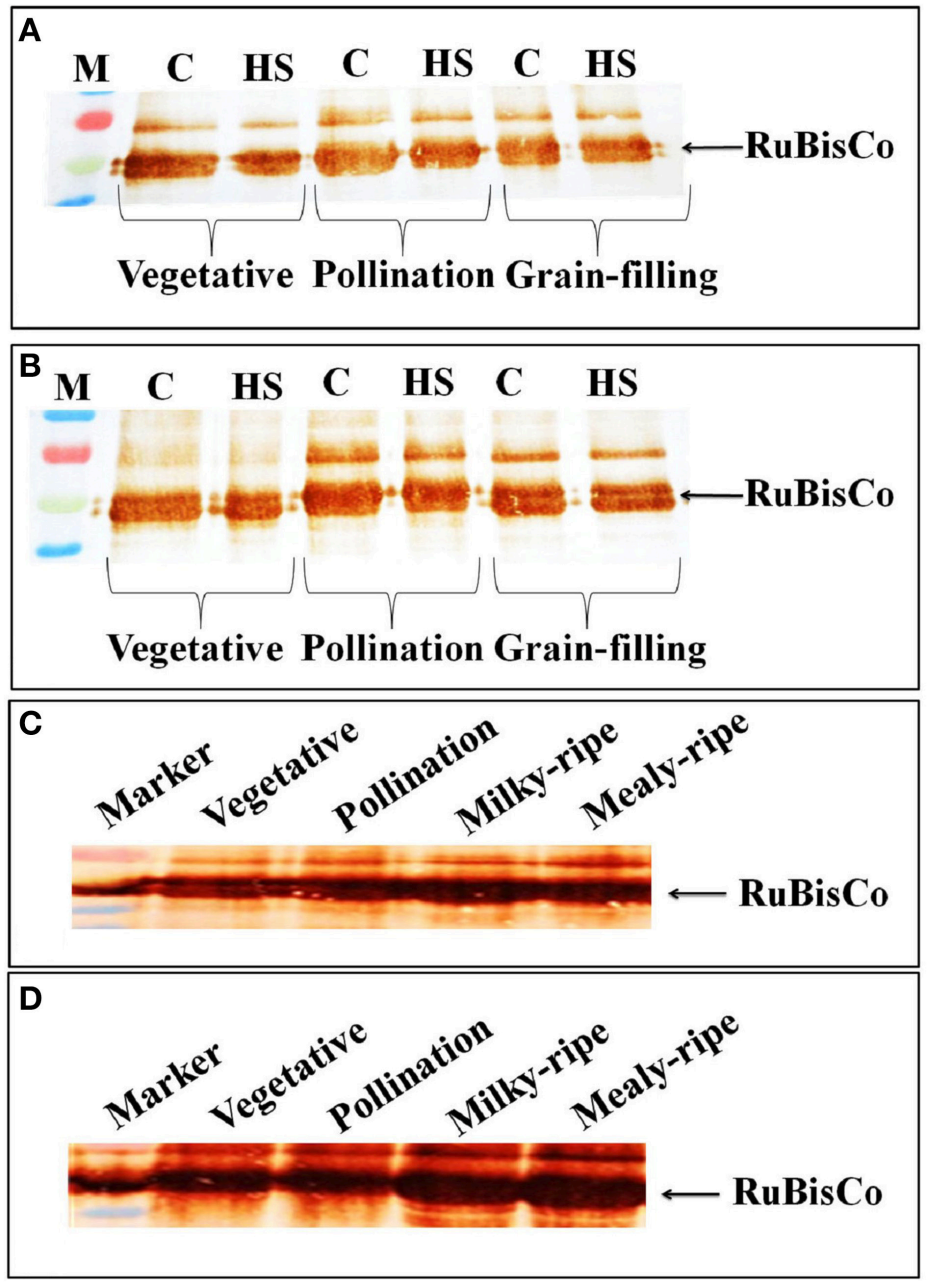
**Accumulation pattern of RuBisCo in wheat at different stages of growth and under heat stress. (A)** RuBisCo accumulation in HD2329 under control and HS, **(B)** RuBisCo accumulation in HD2985 under control and HS, **(C)** RuBisCo accumulation in HD2985 at different stages of growth, **(D)** RuBisCo accumulation in HD2329 at different stages of growth; C −22 ± 3°, heat stress −42°C for 2 h; Polyclonal antibody (anti-RuBisCo) was used along with HRP-conjugated secondary antibody; different letters above each bar indicate a significant difference (*p* < 0.05) between the treatments (one-way ANOVA); vertical bars indicate SE (*n* = 3).

In HD2329, we observed decrease in the intensity of the RuBisCo (Large Subunit) band in response to HS, as compared with the control, at vegetative, pollination, and grain-filling stages; the decrease was more prominent in HS-treated samples at the vegetative stage (Figure 8A). Maximum accumulation of RuBisCo (LSU) was in HD2329 during the pollination stage. In HD2985, the effect of HS on accumulation of RuBisCo (LSU) was not prominent at different stages of growth and only slight decrease in the band intensity was observed under HS (Figure 8B).

Stage-specific RuBisCo accumulation-pattern was also studied in the wheat cultivars during all the stages mentioned above. Prominent accumulation of RuBisCo was observed during the milky-ripe and mealy-ripe sub-stages in HD2985 (Figure 8C), whereas in HD2329, maximum accumulation was observed during the pollination and milky-ripe stage, which decreased thereafter (Figure 8D).

### Pattern of sugars accumulation in stems and leaves under HS

**Figure 9:**
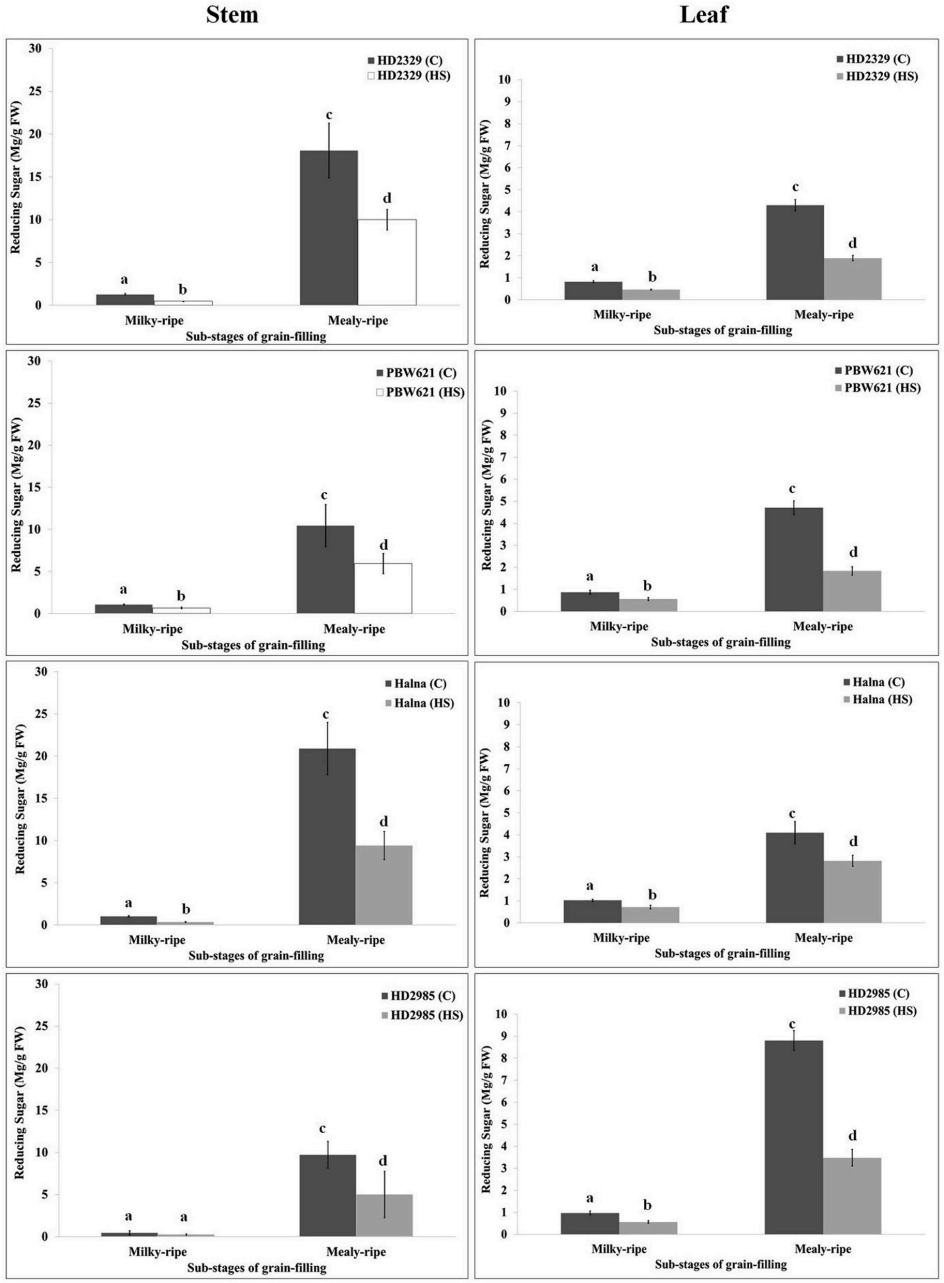
**Variations in the reducing sugar content in stem and leaves of contrasting wheat ***cvs***. under control and heat stress conditions**. HD2329, PBW621, Halna, and HD2985 were used for the reducing sugar pattern profiling; C −22 ± 3°, heat stress −42°C for 2 h; Milky-ripe and mealy-ripe sub-stages of grain-filling were used for the analysis; different letters above each bar indicate a significant difference (*p* < 0.05) between the treatments (one-way ANOVA); vertical bars indicate SE (*n* = 3).

RuBisCo and Rca directly influence the synthesis and distribution of reducing and non-reducing sugars in the leaves (source), stems, and spikes (sink); however, carbon partitioning and stem-reserve mobilization varies with the environmental temperature. HD2329 showed maximum accumulation of reducing sugar in stem during mealy-ripe stage (18 mg/g fresh weight) under control condition. HS causes drastic reduction in the accumulation of reducing sugar in stem of HD2329 during mealy-ripe stage (Figure 9). Similar pattern of reducing sugar accumulation was observed in stems of other cultivars (HD2985, PBW621, and Halna) in response to control and HS-treated conditions (Figure 9). Halna showed maximum accumulation of reducing sugar (21.5 mg/g fresh weight) in stem during mealy-ripe stage, as compared to other *cvs*. under control condition. Similarly, under HS, stem of HD2329 showed maximum accumulation of reducing sugar (10 mg/g FW) compared to other cultivars.

Reducing sugar estimation in leaves of wheat *cv*. HD2329 showed maximum accumulation during mealy-ripe stage under control (4.4 mg/g FW) and HS-treated (2.1 mg/g FW) conditions. Similar pattern of accumulation of reducing sugar was observed in other wheat *cvs*. at different sub-stages of grain-filing under control and HS-treated conditions. Leaves of HD2985 showed maximum accumulation of reducing sugar during mealy-ripe stage under control and HS-treated conditions, as compared to other cultivars (Figure 9).

**Figure 10:**
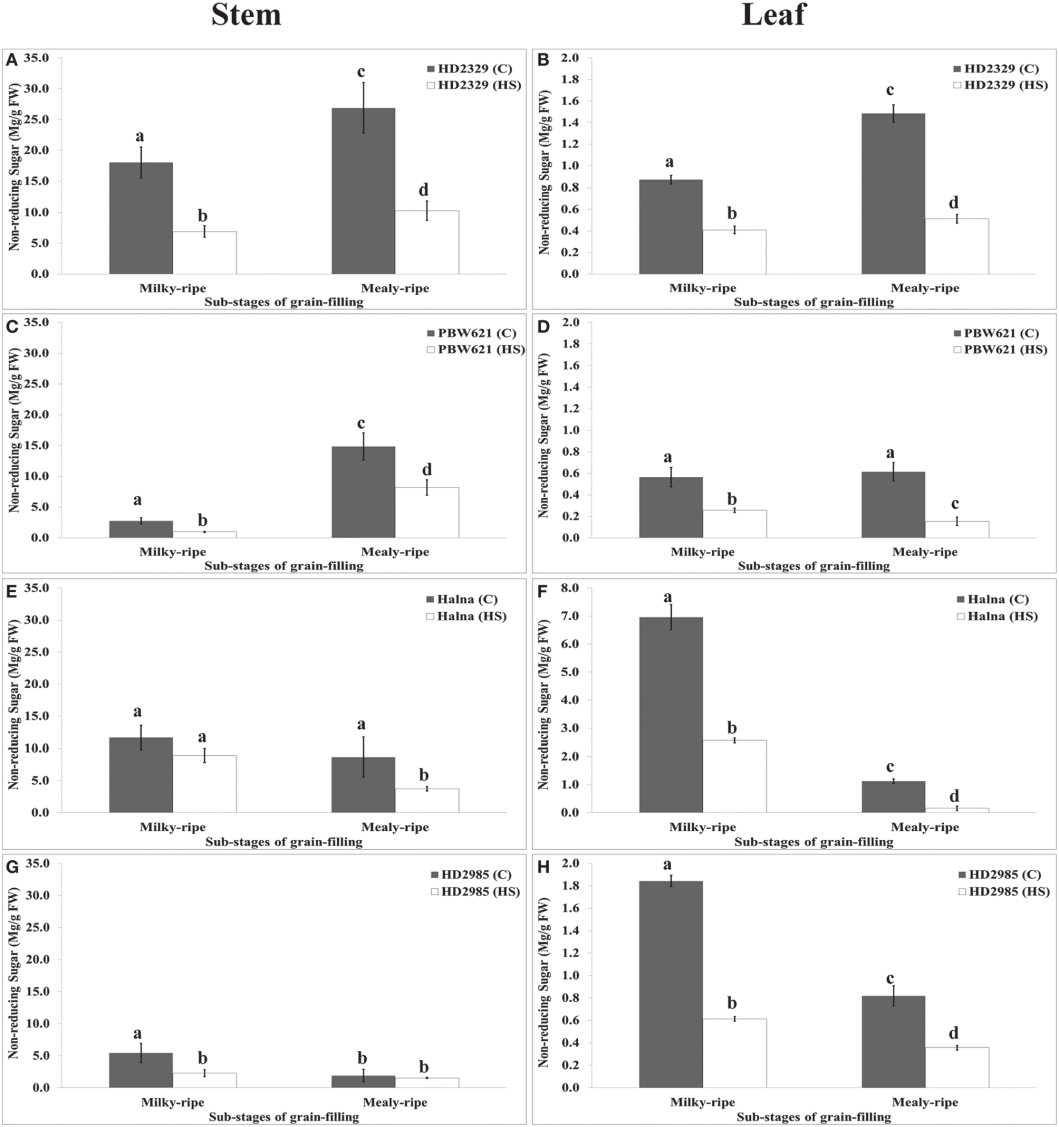
**Changes in the non-reducing sugar accumulation in stem and leaves of wheat ***cvs***. under control and heat stress conditions**. **(A,B)** Non-reducing sugar content in stem and leaf of HD2329, **(C,D)** Non-reducing sugar accumulation in stem and leaf of PBW621, **(E,F)** Non-reducing sugar content in stem and leaf of Halna, **(G,H)** Non-reducing sugar accumulation in stem and leaf of HD2985. Thermosusceptible (HD2329 and PBW621) and thermotolerant (Halna and HD2985) were used for the non-reducing sugar accumulation pattern analysis; C −22 ± 3°, heat stress –42°C for 2 h; Milky-ripe and mealy-ripe sub-stages of grain-filling were used for the analysis; different letters above each bar indicate a significant difference (*p* < 0.05) between the treatments (one-way ANOVA); vertical bars indicate SE (*n* = 3).

HD2329 showed maximum accumulation of non-reducing sugar in stem during mealy-ripe under control (27 mg/g FW) condition, which drastically decrease under HS (10 mg/g FW) (Figure 10). Similar pattern of non-reducing sugar accumulation was observed in stem of PBW621 under control and HS-treated conditions. Halna showed maximum accumulation of non-reducing sugar in stem during milky-ripe stage (11.5 mg/g FW) under control condition (Figure 10). We observed significant decrease in the non-reducing sugar accumulation in stem of PBW621 under HS at different sub-stages of grain-filling. Non-reducing sugar accumulation was observed minimum in stem of HD2985 compared to other *cvs*., both at milky- and mealy-ripe stages under control and HS-treated conditions. Non-reducing sugar content in leaves of HD2329 showed maximum accumulation during mealy-ripe stage under control and HS-treated conditions. Non-significant variations in the non-reducing sugar accumulation were observed in PBW621 under control and HS-treated conditions. Halna showed maximum accumulation of non-reducing sugar in leaves during milky-ripe, both under control and HS-treated conditions, which reduces subsequently during mealy-ripe stage. Similar pattern of non-reducing sugar accumulation in leaves of HD2985 was observed under control and HS-treated conditions (Figure 10). Non-reducing sugar in leaves of HD2985 decreased significantly under the control and HS-treated conditions during the grain-filling; decrease was more during the milky-ripe stage.

### Variation in the total antioxidant capacity (TAC) under HS

**Figure 11:**
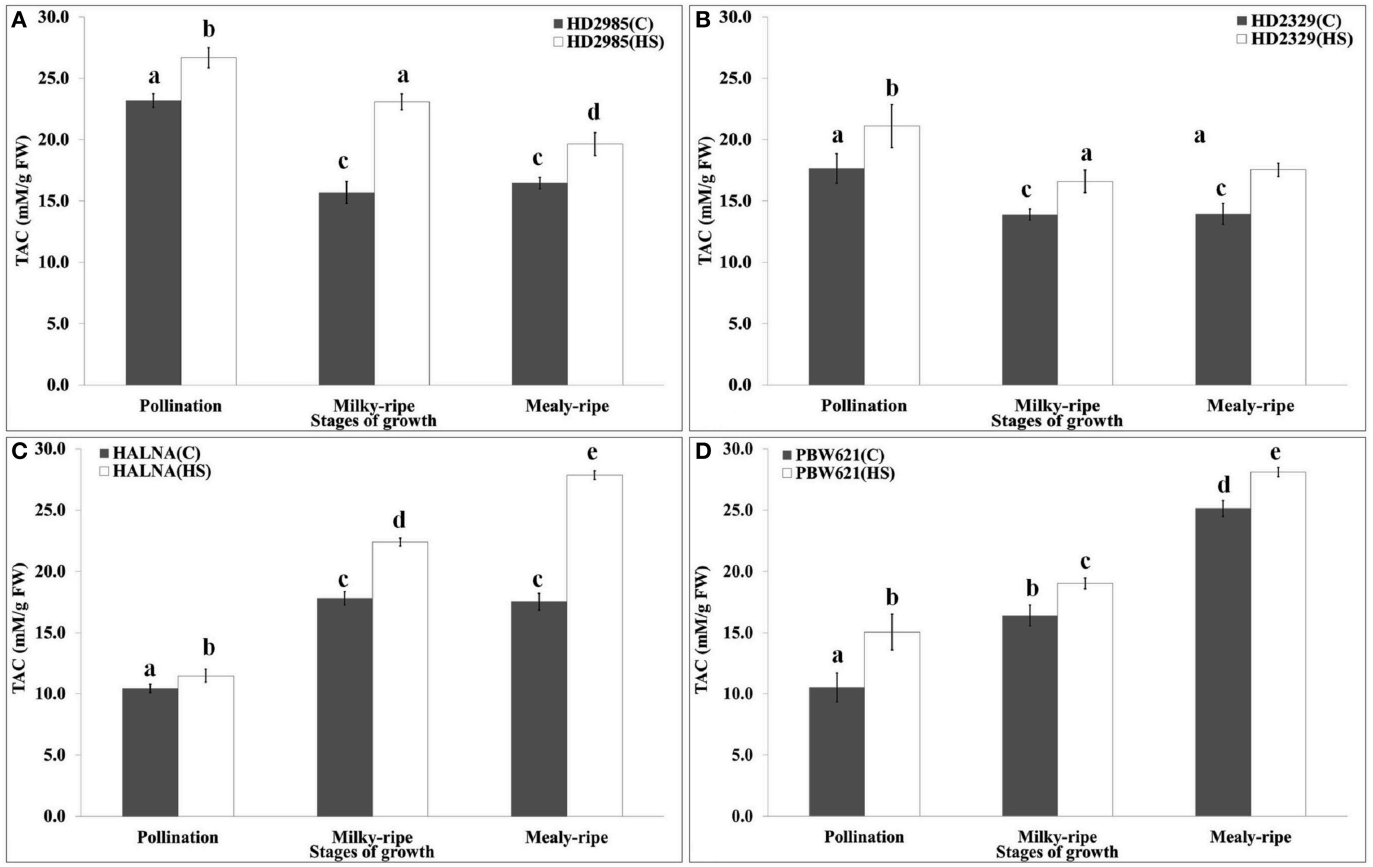
**Total Antioxidant Capacity (TAC) profiling of contrasting wheat cultivars at different stages of development and under the heat stress. (A)** TAC in HD2985, **(B)** TAC in HD2329, **(C)** TAC in Halna, **(D)** TAC in PBW621; Samples collected from the pollination, milky-ripe and mealy-ripe stages were used for the TAC estimation; C −22 ± 3°, heat stress −42°C for 2 h; vertical bars indicate SE (*n* = 3).

TAC is a measure of the antioxidant potential of the tissues, and is one of the important biochemical parameters used for assessing the thermotolerance of crops. We observed significant decrease in the TAC in HD2985 during mealy-ripe, as compared to pollination and milky-ripe stages (Figure 11). An increase in the TAC was observed in response to HS at different stages of development. Similar pattern of TAC was observed in HD2329 (thermosensitive *cv*.) under the control and HS-treated conditions, but TAC was significantly lower than those in HD2985 at all the stages and under different conditions. Significant increase in the TAC, both under control and HS-treated conditions, at different stages of growth were observed in Halna; highest, being during mealy-ripe. We observed significant increase in the TAC in response to HS during pollination, milky-ripe, and mealy-ripe stages of Halna; highest at the mealy-ripe sub-stage. Similar pattern was observed in PBW621; increase in TAC due to HS was quite significant. During the pre-anthesis and milky-ripe stages, highest TAC were observed in HS-treated HD2985 (26.7 and 23 mM/g FW), whereas HS-treated Halna showed highest TAC (27.8 mM/g FW) during the mealy-ripe stage. Maximum increase in the TAC in response to HS was observed in HD2985 during the pollination (25.8%) and milky-ripe (22.3%) and in Halna (27.2%) during the mealy-ripe stage (Figure 11).

### Correlation between the expression and activities of Rca and RuBisCo under HS

**Figure 12:**
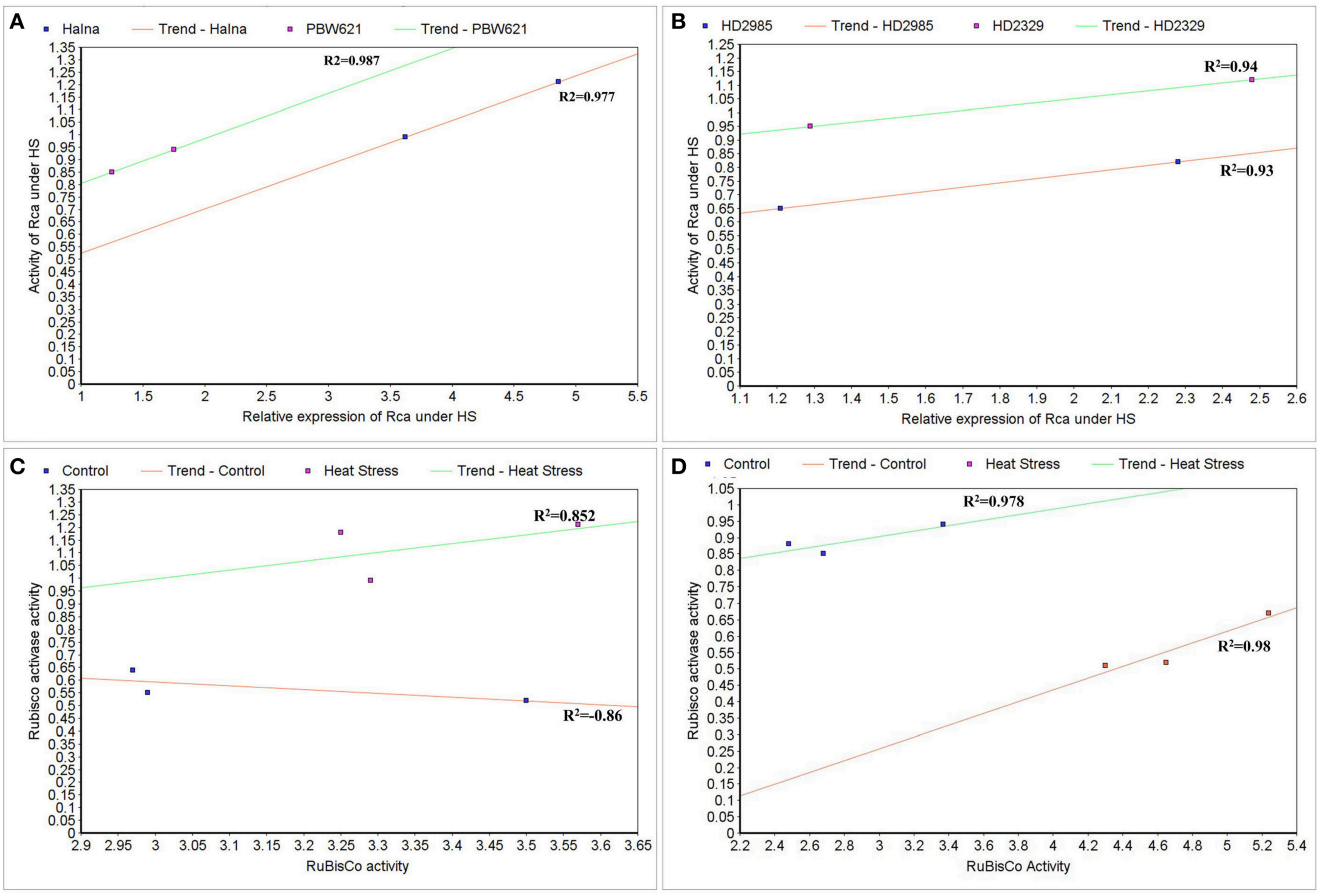
**Cluster-map for analyzing the correlation between expression and activity of RuBisCo activase (Rca) and RuBisCo under control and heat stress conditions. (A)** Correlation between the expression and activity of Rca in Halna and PBW621 (C and HS), **(B)** Correlation between the expression and activity of Rca in HD2985 and HD2329 (C and HS), **(C)** Correlation between the activity of Rca and RuBisCo in Halna (C and HS), **(D)** Correlation between the expression and activity of Rca and RuBisCo in PBW621 (C and HS); C—22 ± 3°, heat stress—42°C for 2 h.

To correlate the transcript level and protein activity of *TaRca1* in Halna, PBW621, HD2985, and HD2329, cluster map analysis was carried out under HS-treated condition. A positive correlation was observed between the relative expression of *TaRca1* and its activity in HS-treated Halna (*R*^2^ = 0.97) and PBW621 (*R*^2^ = 0.98); HS-treated HD2985 and HD2329 showed correlation coefficients of 0.93 and 0.94, respectively (Figures 12A,B).

The Rca activity was also found correlated with the RuBisCo activity in wheat under HS; positive correlation was established in Halna (HS-treated) with *R*^2^ = 0.85 and negative correlation was observed in Halna (control) with *R*^2^ = −0.86. The correlation was further analyzed and confirmed in the thermotolerant wheat *cv*. PBW621—positive correlation in control (0.98) and HS-treated (0.97) was observed (Figures 12C,D).

### Correlation between Rca/RuBisCo activities and photosynthetic rate under HS

**Figure 13:**
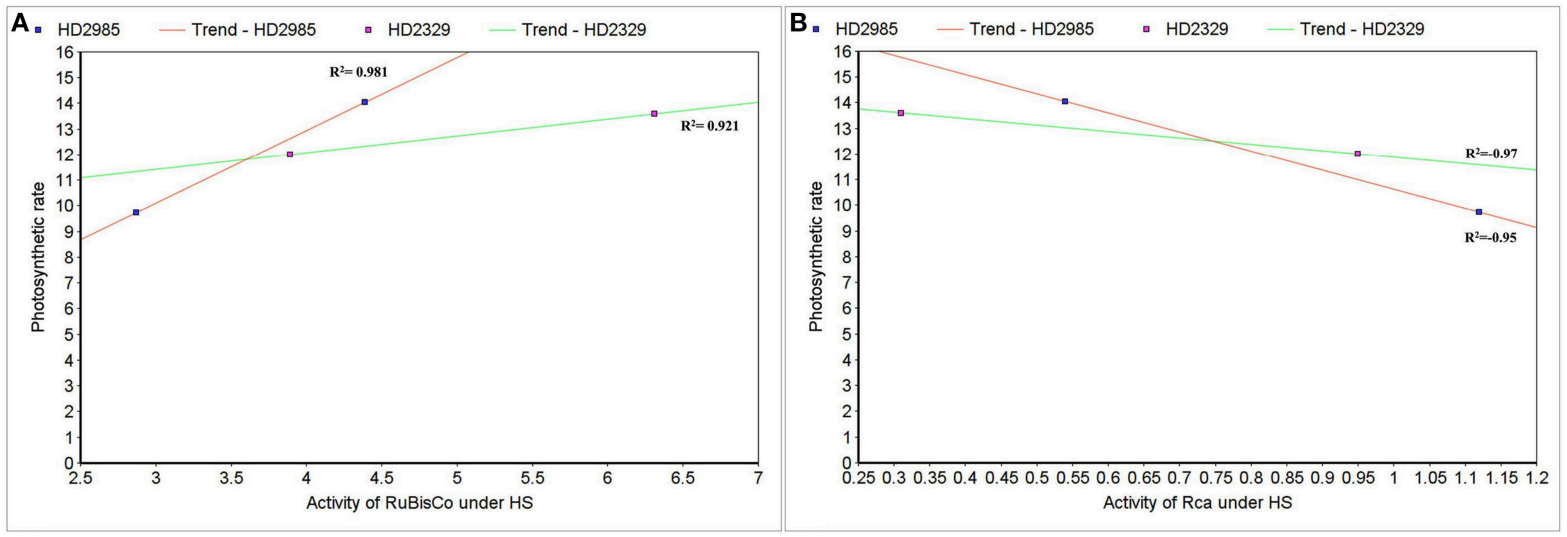
**Correlating the activity of RuBisCo activase (Rca) and RuBisCo with photosynthetic rate in wheat under the heat stress. (A)** Cluster-map analysis of RuBisCo activity with photosynthetic rate in HD2985 and HD2329 under HS, **(B)** Cluster-map analysis of Rca activity with photosynthetic rate in HD2985 and HD2329 under HS.

To establish the relationship of Rca and RuBisCo activities with overall photosynthetic rate of wheat under HS, scatter-plot analysis was carried out and we observed positive correlation between the RuBisCo activity and photosynthetic rate in wheat *cvs*. HD2985 (0.98) and HD2329 (0.92); however Rca showed negative correlation with the photosynthetic rate in both the cultivars with *R*^2^-values of –0.97 and –0.95, respectively (Figures 13A,B).

## Discussion

Wheat is highly-sensitive to HS which results in drastic reduction in photosynthetic rate and translocation of photosynthates from leaves (source) to the endospermic tissues (sink), culminating into significant reduction in the yield. Even slight fluctuation in the temperature during the critical stages such as pollination and grain-filling has been reported to significantly reduce the yield of wheat.

We identified eight transcripts of *Rca* by mining the RNA-seq data and cloned a full-length putative *Rca* transcript of 1402 nt with an open reading frame of 332 aa. Law and Crafts-Brandner (1999) have reported two isoforms of *Rca*—α-isoform (46 kDa) and β-isoform (42 kDa)—in leaves of wheat. Using the genome database for the Chinese spring wheat (IWGSC, 2014), two *Rca* sequences were identified in wheat with different isoforms—*TaRca-1* and *TaRca2*; the genes were cloned and submitted to EMBL (http://www.ebi.ac.uk/ena/) under the accession numbers LM992844 (*TaRca1-*β), LM992845 (*TaRca2-*β), and LM992846 (*TaRca2-*α). Genome-wide survey revealed the presence of α- and β-form *Rca* in the maize genome (*ZmRCA*α and *ZmRCA*β; Yin et al., 2014). Southern blot analysis showed the presence of single copy of the cloned *TaRca1* gene in wheat which is in conformity with the observation of Law and Crafts-Brandner (1999), who reported the presence of single copy number of *Rca* gene in wheat; similar findings were reported by To et al. (1999) in *Oryza sativa*.

We observed significant increase in the expression of Rca in different wheat *cvs*. under HS. Transcriptional analyses showed specific diurnal pattern of expression of *ZmRCA*α and *ZmRCA*β; abundance was observed higher in leaves and grains (Yin et al., 2014). Feng et al. (2007) observed increase in the accumulation of *Rca* in the transgenic rice in response to HS, as compared to the wild plants. Diurnal changes in the expression of three isoforms of *Rca* were observed in cotton under HS (DeRidder and Salvucci, 2007). Very high accumulation of *RcaL* isoform, as compared with that of *RcaS*, was observed in the leaves and green stems of *Brachypodium distachyon*, under the salt and drought stress, respectively (Bayramov and Guliyev, 2014). Alternative splicing has been reported to play a role in the post-transcriptional regulation of *Rca*, in response to different growth and stress conditions (DeRidder et al., 2012). A significant, positive, and linear correlation has been reported between the expression of wheat 45–46 kDa Rca and plant productivity, under the heat-stress condition in wheat (Ristic et al., 2009). Here, we observed that heat stress significantly induces the expression and activity of Rca, which is in conformity with the report of Wang D. et al. (2010) in rice.

We observed increase in the activity of Rca in response to HS in the contrasting wheat *cvs*.; highest activity was observed in the thermotolerant cultivars (Halna and HD2985) during the mealy-ripe stage. Rokka et al. (2001) have reported temperature-dependent dual role of Rca—removing inhibitory sugar phosphates under the normal ambient temperatures, and acting as chaperone along with thylakoid-bound ribosomes, under the sudden outburst of temperature (HS). Salvucci et al. (2001) studied thermal stability of Rca *in vivo* and *in vitro*, and reported that the activase exhibits a temperature optimum of 44°C for ATP hydrolysis as compared to ≥60°C for carboxylation by RuBisCo. Salvucci and Crafts-Brandner (2004a) reported decreased ability of Rca to activate RuBisCo *in vitro* under HS. The transcript abundance and protein expression levels of *Rca* genes were found to be positively correlated with grain yield in 128 maize inbred lines (Yin et al., 2014). We observed increase in the RuBisCo proteins in response to HS in all the *cvs*. The finding is in conformity with the observation of Demirevska-Kepova et al. (2005) in wheat under differential HS.

We observed decrease in the activity of RuBisCo under HS; the decrease may be due to less availability of the activated enzyme under HS (Galmes et al., 2013). The rate of deactivation of RuBisCo was observed to increase with increase in the temperature (Salvucci and Crafts-Brandner, 2004c). The activation state of RuBisCo was reported to decrease, along with the inhibition of Rca, in HS-treated cotton and extent of RuBisCo deactivation was correlated with the metabolic limitation to photosynthesis (Carmo-Silva et al., 2012). Salvucci and Crafts-Brandner (2004c) showed that RuBisCo was inactivated within 7 s of imposing a heat stress in cotton and tobacco due to decrease in the activase activity.

Under the present investigation, we observed increase in the accumulation of RuBisCo protein at different stages of growth under the control; however, decrease in RuBisCo accumulation was observed in response to HS (38°C, 2 h). The finding is in conformity with the observation of Demirevska-Kepova et al. (2005) who reported decrease in the RuBisCo protein under HS in wheat. Similar observations have been reported in response to different stresses in plants (Wang D. et al., 2010; Galmes et al., 2013) observed through immunoblot analysis that the ratio of RCA_L_ to RuBisCo increased significantly in heat-acclimated rice leaves.

An alteration in RuBisCo and Rca activities under HS causes change in the carbon assimilatory processes, which is evident from the increased level of non-reducing sugar in the leaves and stem. Stem-reserves have been recognized as an important source of carbon for grain-filling, when photosynthesis is inhibited by the abiotic stresses such as heat and drought (Blum, 1998). Accumulation of sugars in stem has been predicted to be an adaptive strategy against different stresses, as found in different genotypes of sorghum under the drought stress (Quazi et al., 2014). Asthir and Bhatia (2014) have reported that HS causes built-up of total free sugars and decreases the starch content of grains in wheat. Similar finding were reported by Kumar and Rai (2014). Liu and Huang (2000) have also observed decrease in the total non-structural carbohydrates (TNC), fructans, starch, glucose, and sucrose in shoots (leaves and stems) and roots in response to HS in the bentgrass cultivars. Increase in the TAC under HS with greater increase in thermotolerant is in agreement with the findings of Chakrabortty and Pradhan (2011) that the activities of the antioxidant enzymes involved in thermotolerance goes up under HS; similar observations were reported by us also Kumar et al. (2013) and Kumar and Rai (2014).

Photosynthetic rate decreased under HS in all the cultivars, except in PBW621 where increase in the P_n_ was observed. Farooq et al. (2011) reported reduction in the photosynthetic capacity of the plant under HS, mainly due to the oxidative damage to the chloroplasts, causing reductions in the dry matter accumulation and grain yield. Similarly, Wang G.P. et al. (2010) observed increase in the transpiration rate (T_r_), stomatal conductance (G_s_), and intercellular CO_2_ concentration (C_i_), and decrease in the photosynthetic rate in wheat under heat stress.

Yamori et al. (2012) reported that HS of 40°C caused decrease in the accumulation of Rca, which in turn reduced the RuBisCo activation state and steady-state photosynthesis, is concordant with the findings of this study. Higher RuBisCo activation and CO_2_ assimilation were observed in rice, with over-expressed Rca (Fukayama et al., 2012). Here, we observed negative correlation between the Rca and photosynthetic rate of wheat under HS. Sage et al. (2008) reported that Rca, being highly heat-labile, shown to limit the photosynthesis under the slightest fluctuation in temperature. Similar finding was reported by Salvucci and Crafts-Brandner (2004b) in wheat under HS. Similarly, positive correlation of P_n_ with the RuBisCo activity and negative with Rca activity under the HS, as observed in present investigation is contrary to the findings of Wang G. P. et al. (2010) who reported positive correlation between the Rca protein content, RuBisCo initial activity and net photosynthetic rate (Pn) under normal and HS conditions; dual role of Rca under the ambient and HS conditions may be one of the reason (Rokka et al., 2001). Negative correlation between RuBisCo and Rca observed in Halna could not be explained.

## Conclusion

We identified eight putative transcripts of *Rca* by mining NGS data and cloned a novel TaRca1 gene of 1402 bp from wheat *cv*. HD2985. Single copy of the cloned *TaRca1* was observed in wheat genome. Expression and activity of TaRCa1 was observed maximum in Halna during pollination and grain-filling under control and HS, as compared to HD2985, HD2329, and PBW621. An increase in the RuBisCo activity was observed in Halna during grain-filling under HS. We observed negative correlation between the RuBisCo activity and protein accumulation, as validated through Immunoblot analysis under control and HS conditions. A positive correlation was established between the expression and activity of RuBisCo and *TaRca1* under HS; proper distributions of reducing and non-reducing sugars were observed in cultivars having high activity of RuBisCo and Rca. TAC was observed higher in *cv*. with maximum RuBisCo and Rca activities. TaRca1 has the potential to modulate the activity of RuBisCo under HS and provides the most probable solution for mitigating the HS problem in wheat.

## Author contributions

RK and SG conceived and designed the experiment; KS, KD, RS, and YK performed the heat stress treatment, sample collection, activity assay, and Real-Time PCR; NV, SS, DM, MG, and AR performed the data analysis; BS and GR, performed the RuBisCo and Rca activity assay; HP and VC executed the IRGA experiment and correlated the derived results; RR, SG, SP, and VC wrote the manuscript; all authors contributed to the discussion and approved the final manuscript.

### Conflict of interest statement

The authors declare that the research was conducted in the absence of any commercial or financial relationships that could be construed as a potential conflict of interest.

## Supplementary material

Figure S1 **Work-plan depicting the layout for the sowing, heat shock treatment and samples collection at different stages of growth and development for downstream analysis**.

Table S1 **List of putative RuBisCo activase genes identified by mining of ***de novo*** transcriptome data generated from control and heat shock-treated wheat ***cv***. HD2985 using next-generation sequencing (NGS) platform**.

Table S2 **Identification of putative RuBisCo activase (***Rca***) transcript sequences from control and HS-treated wheat ***cv***. HD2985 using RNA-seq**.

Table S3 **List of primers used for the cloning and expression study of RuBisCo activase (***TaRca1***) by quantitative real-time PCR (qRT-PCR)**.
