# Supplementary figures and images for: Identification of Putative RuBisCo Activase (TaRca1)—The Catalytic Chaperone Regulating Carbon Assimilatory Pathway in Wheat (Triticum aestivum) under the Heat Stress
Source: Front Plant Sci. 2016 Jul 12;7:986. doi: 10.3389/fpls.2016.00986 (PMC4940427; doi:10.3389/fpls.2016.00986)

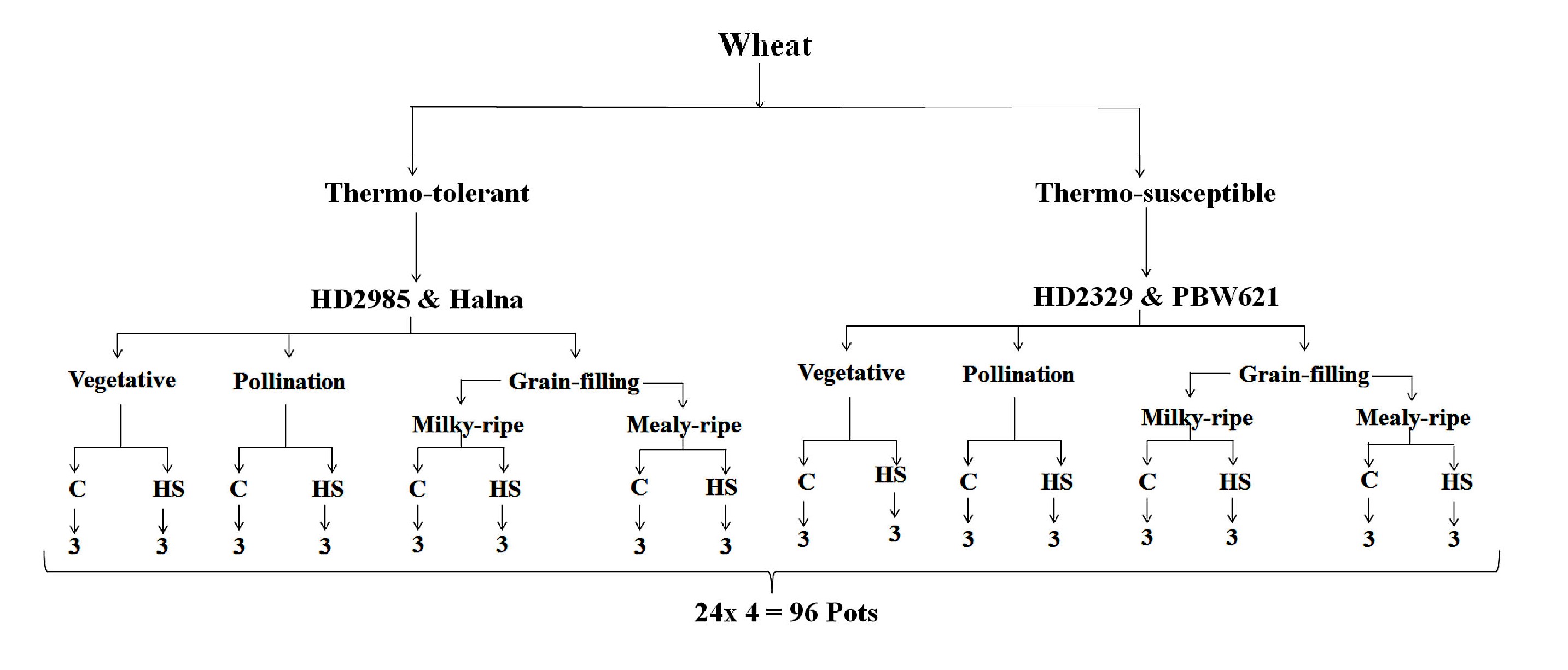

Supplement: Figure S1 — Work-plan depicting the layout for the sowing, heat shock treatment and samples collection at different stages of growth and development for downstream analysis. [file Image1.jpg]
