# Supplementary material for: Identification of Putative RuBisCo Activase (TaRca1)—The Catalytic Chaperone Regulating Carbon Assimilatory Pathway in Wheat (Triticum aestivum) under the Heat Stress
Source: Front Plant Sci. 2016 Jul 12;7:986. doi: 10.3389/fpls.2016.00986 (PMC4940427; doi:10.3389/fpls.2016.00986)
Supplement: Table S1 — List of putative RuBisCo activase genes identified by mining of de novo transcriptome data generated from control and heat shock-treated wheat cv. HD2985 using next-generation sequencing (NGS) platform. [file Table1.docx]

**Table S1** List of Rubisco activase (Rca) primers used for the cloning and expression study by quantitative real-time PCR (qRT-PCR)

| Primer ID | Primers sequence (5’-3’) | Tm (ºC) |
| --- | --- | --- |
| TaRca-F | GCATCGCATCTGTACTTGCTAG | 56.5 |
| TaRca-R | GGTTACCTGTGCGGCGATG | 56.0 |
| qTaRca-f | TACGACATCTCCGATGACCA | 60.0 |
| qTaRca-r | CTCGTAGGAGCTCAGGATGG | 59.9 |
| Act-f | GCGGTCGAACAACTGGTATT | 58.4 |
| Act-r | GGTCCAAACGAAGGATAGCA | 58.4 |

*q- Quantitative; F – Forward; r – Reverse; Ta – *Triticum aestivum*
