# Supplementary material for: Identification of Putative RuBisCo Activase (TaRca1)—The Catalytic Chaperone Regulating Carbon Assimilatory Pathway in Wheat (Triticum aestivum) under the Heat Stress
Source: Front Plant Sci. 2016 Jul 12;7:986. doi: 10.3389/fpls.2016.00986 (PMC4940427; doi:10.3389/fpls.2016.00986)
Supplement: Table S2 — Identification of putative RuBisCo activase (Rca) transcript sequences from control and HS-treated wheat cv. HD2985 using RNA-seq. [file Table2.docx]

**Table S2** List of putative RuBiCo activase genes identified by mining of *De novo* transcriptome data generated from control and heat stress-treated wheat *cv*. HD2985 using next-generation sequencing platform

| **Sample Name** | **Domain Families** | **Transcript Length** | **Peptide Length** | **Hit_Acc.** | **Alignment Length** | **Start Position** | **End Position** | **Chromosomal localization** |
| --- | --- | --- | --- | --- | --- | --- | --- | --- |
| **H2985_Control** |  |  |  |  |  |  |  |  |
| Transcript_13 | AAA superfamily | 1222 | 408 | PLN00020 | 350 | 2 | 352 | 5B |
| Transcript_21 | AAA superfamily | 1045 | 349 | PLN00020 | 273 | 15 | 288 | 5B |
| Transcript_33 | AAA superfamily | 1039 | 347 | PLN00020 | 273 | 15 | 288 | 5B |
| **H2985_Stress** |  |  |  |  |  |  |  |  |
| Transcript_5 | AAA superfamily | 1402 | 468 | PLN00020 | 412 | 1 | 412 | 7A |
| Transcript_8 | AAA superfamily | 1228 | 440 | PLN00020 | 377 | 1 | 377 | 5B |
| Transcript_11 | AAA superfamily | 1252 | 420 | PLN00020 | 364 | 1 | 364 | 5B |
| Transcript_19 | AAA superfamily | 1237 | 413 | PLN00020 | 326 | 2 | 328 | 5B |
| Transcript_22 | AAA superfamily | 1237 | 413 | PLN00020 | 326 | 2 | 328 | 5B |
