# Supplementary material for: Identification of Putative RuBisCo Activase (TaRca1)—The Catalytic Chaperone Regulating Carbon Assimilatory Pathway in Wheat (Triticum aestivum) under the Heat Stress
Source: Front Plant Sci. 2016 Jul 12;7:986. doi: 10.3389/fpls.2016.00986 (PMC4940427; doi:10.3389/fpls.2016.00986)
Supplement: Table S3 — List of primers used for the cloning and expression study of RuBisCo activase (TaRca1) by quantitative real-time PCR (qRT-PCR). [file Table3.docx]

**Table S3** Identification of putative RuBisCo activase (Rca) transcript sequences from control and HS-treated wheat *cv*. HD2985 using RNA-seq

| HD2985_Control | Nucleotides |
| --- | --- |
| >transcript_13  atggcagcggaaaacctcgacgagaagaggaacacggacaagtggaagggtcttgcgtacgatatctccgacgaccagcaggacatcaccagagggaagggcatcgtggactcgctcttccaggcgcccacgggcgacggcacccacgaggccgtcctcagctcctacgagtacgtcagccagggactcaagaagtacgacttcgacaacaccatgggaggcttctacatcgctcctgctttcatggacaagcttgttgtccatctctccaagaacttcatgaccctgcccaacatcaagatcccactcatcttgggtatctggggagcaaggatgggcatcaacccaatcatgatgagtgccggagagctggagagtggcaacgccggagagccagccaagctcatcaggcagcggtaccgtgaggctgcagacatgatcaagaagggtaagatgtgctgcctcttcatcaacgatcttgacgctggtgcgggtcggatgggcgggaccacacagtacaccgtcaacaaccagatggtgaacgccaccctcatgaacatcgccgatgcccccaccaacgtgcagctcccaggcatgtacaacaaggaggagaaccctcgtgtgcccatcgtcgtcactggtaacgatttctcgacgttgtacgcccctctgatccgtgatggtcgtatggagaagttctactgggctcccacccgcgacgaccgtatcggtgtctgcaagggtatcttccagaccgacaatgtcagcgacgagtccgtcgtcaagatcgtcgacaccttcccaggacaatccatcgactttttcggtgctctgcgtgctcgggtgtacgacgacgaggtgcgcaagtgggtgacctctaccggtatcgagaacattggcaagaagctggtgaactcgcgggacggaccagtgacctttgagcagccaaagatgacagtggagaagctgctagagtacgggcacatgctcgtccaggagcaggacaatgtcaagcgtgtgcagcttgctgacacctacatgagccaggcagctctgggtgatgctaaccaggatgcgatgaagactggtaccttctacggaggaggtaaaggggcacagcaaggtactttgcctgtaccggcaggatgcaccgaccagactgccaagaacttcgacccaacggcgaggagtgacgacggcagctgcctttacaccttttaa | 1221 |
| >transcript_21  atgatgagtgccggagagctggagagtggcaacgccggagagctcatgaccctgcccaacatcaagatcccactcatcttgggtatctggggaggcaagggtcaaggaaaatccttccagtgtgagcttgtcttcgccaagatgggcatcaacccaatcatgatgagtgccggagagctggagagtggcaacgccggagagccagccaagctcatcaggcagcggtaccgtgaggctgcagacatgatcaagaagggtaagatgtgctgcctcttcatcaacgatcttgacgccggtgcgggtaggatgggcgggaccacacagtacaccgtcaacaaccagatggtgaacgccaccctcatgaacatcgccgatgcccccaccaacgtgcagctcccaggcatgtacaacaaggaggagaaccctcgtgtgcccatcgtcgtcactggtaacgatttctcgacgttgtacgcccctctgatccgtgatggtcgtatggagaagttctactgggctcccacccgcgacgaccgtatcggtgtctgcaagggtatcttccagaccgacaatgtcagcgacgagtccgtcgtcaagatcgtcgacaccttcccaggacaatccatcgactttttcggtgctctgcgtgctcgggtgtacgacgacgaggtgcgcaagtgggtgacctctaccggtatcgagaacattggcaagaggctggtgaactcgcgggacggacccgtgacctttgagcagccaaagatgacagtggagaagctgctagagtacgggcacatgctcgtccaggagcaggacaatgtcaagcgtgtgcagcttgctgacacctacatgagccaggcagctctgggtgatgctaaccaggatgcgatgaagactggttccttctacggaggaggtaaaggggcacagcaaggtactttgcctgtaccggcaggatgcaccgaccagactgccaagaacttcgacccaacggcgaggagtgacgacggcagctgcctttacacccttttaagcatgccaatttaa | 1044 |
| >transcript_33  atgatgagtgccggagagctggagagtggcaacgccggagagctcatgaccctgcccaacatcaagatcccactcatcttgggtatctggggaggcaagggtcaaggaaaatccttccagtgtgagcttgtcttcgccaagatgggcatcaacccaatcatgatgagtgccggagagctggagagtggcaacgccggagagccagccaagctcatcaggcagcggtaccgtgaggctgcagacatgatcaagaagggtaagatgtgctgcctcttcatcaacgatcttgacgccggtgcgggtaggatgggcgggaccacacagtacaccgtcaacaaccagatggtgaacgccaccctcatgaacatcgccgatgcccccaccaacgtgcagctcccaggcatgtacaacaaggaggagaaccctcgtgtgcccatcgtcgtcactggtaacgatttctcgacgttgtacgcccctctgatccgtgatggtcgtatggagaagttctactgggctcccacccgcgacgaccgtatcggtgtctgcaagggtatcttccagaccgacaatgtcagcgacgagtccgtcgtcaagatcgtcgacaccttcccaggacaatccatcgactttttcggtgctctgcgtgctcgggtgtacgacgacgaggtgcgcaagtgggtgacctctaccggtatcgagaacattggcaagaggctggtgaactcgcgggacggacccgtgacctttgagcagccaaagatgacagtggagaagctgctagagtacgggcacatgctcgtccaggagcaggacaatgtcaagcgtgtgcagcttgctgacacctacatgagccaggcagctctgggtgatgctaaccaggatgcgatgaagactggttccttctacggtaaaggggcacagcaaggtactttgcctgtgccggcaggatgcaccgaccagactgccaagaacttcgacccaacggcgaggagtgacgacggcagctgcctttacacccttttaagcatgccaatttaa | 1038 |
| HD2985_Stress |  |
| >Transcript_5  cggggagagaacaatcgacacgatggcttctgctttctcgtccaccgttggagctccggcgtcgaccccgaccaccttcctcgggaagaaggtgaagaagcaggccggtgcgttgaactactaccatggtggcaacaagatcaacaatagggtggtcagggccatggcggccaaaaaggaacttgacgagggcaagcagaccgatgccgatcggtggaagggtctcgcttacgacatctccgatgaccagcaggacatcacgagggggaaaggcatcgtggactccctgttccaggcccccatgggcgacggcacccacgaggccatcctgagctcctacgagtacatcagccagggcctgcgcaagtacgacttcgacaacaccatggacgggctgtacatcgccccggcgttcatggacaagctcatcgtccacctcgccaagaacttcatgacactccccaacatcaaggtccctctcatcctgggtatctggggaggcaagggacagggcaagtcgttccagtgcgagctggtgttcgccaagatgggcatcaaccccatcatgatgagcgccggagagctggagagcggcaacgccggcgagccggccaagctgatccggcagaggtaccgcgaggctgccgacattatcaagaagggcaagatgtgctgcctcttcatcaacgacctggacgccggcgcggggcggatgggcgggacgacgcagtacacggtgaacaaccagatggtgaacgccaccctgatgaacatcgcggacgcgcccaccaacgtgcagttcccggggatgtacaacaaggaggagaacccacgcgtgcccatcatcgtcaccggcaacgacttctcgacgctgtacgcgcccctcatccgggacggccgcatggagaagttctactgggcgcccacccgggaggaccgcatcggcgtgtgcaagggcatcttccgcaccgacaacgtccccgacgaggccgtggtgaggctggtggacaccttcccggggcagtccatcgacttcttcggcgcgctgcgggcgcgggtgtacgacgacgaggtgcgcaagtgggtcggcgagatcggcgtcgagaacatctccaagcggctcgtcaactccagggaggggccgccgacgttcgaccagcccaagatgaccatcgagaagctcatggagtacggccacatgctggtccaggagcaggagaacgtgaagcgcgtgcagctcgccgacaagtacctcagcgaggcggcgctcggccaagccaacgacgacgccatggcgaccggcgccttctacggcaagtagaaagtcctatacttaagatgcatgcgtgcatgcatgcactatatatatgctggaatattttggactcgaatccactcaaac | 1402 |
| >transcript_8  atggctgctgccttctcctccaccgtcggtgccccggcttctacgccgaccaacttccttgggaagaagctcaagaagcaggtgacctcggccgtgaactaccatggcatgagctccaaggccaacaggttcacagtcatggcagcggaaaacctcgacgagaagaggaacacggacaagtggaagggtcttgcgtacgatatctccgacgaccagcaggacatcaccagagggaagggcatcgtggactccctcttccaggcgcccacgggcgacggcacccacgaggccgtcctcagctcctacgagtacgtcagccagggactcaagaagtacgacttcgacaacaccatgggaggcttctacatcgctcctgctttcatggacaagcttgttgtccatctctccaagaacttcatgaccctgcccaacatcaagatcccactcatcttgggtatctggggaggcaagggtcaaggaaaatccttccagtgtgagcttgtcttcgccaagatgggcatcaacccaatcatgatgagtgccggagagctggagagtggcaacgccggagagccagccaagctcatcaggcagcggtaccgtgaggctgcagacatgatcaagaagggtaagatgtgctgcctcttcatcaacgatcttgacgccggtgcgggtcggatgggcgggaccacacagtacaccgtcaacaaccagatggtgaacgccaccctcatgaacatcgccgatgcccccaccaacgtgcagctcccaggcatgtacaacaaggaggagaaccctcgtgtgcccatcgtcgtcactggtaacgatttctcaacgttaagtcctactgggcttccaacccgcgacgaccgtatcggtgtctgcaagggtatcttccagaccgacaatgtcagcgacgagtccgtcgtcaagatcgtcgacaccttcccaggacaatccatcgaccttttccggtgctctgcgtgctcgggtgtacgacgacgagtgacctttgagcagccaaagatgacagacgagaagctgctagagtacgggcagatgcttgtccaggagcaggccaatgtcaagcgtgtgcagcttgctgacacctacatgagccaggcagctctgggtgatgctaaccaggatgcgatgaagactggttccttctacggtaaagggacacagcaagactgccaagaacttcgacccaacggcgaggagtga | 1227 |
| >transcript_11  atggctgctgccttctcctccaccgtcggtgccccggcttctacgccgaccaacttcctcgggaagaagctcaagaagcaggtgacctcggccgtgaactaccatggcatgagctccaaggccaacaggttcacagtcatggcagcggaaaacatcgacgagaagaggaacacggacaagtggaagggtcttgcgtacgatatctccgatgaccagcaggacatcaccagagggaagggcatcgtggactccctcttccaggcgcccacgggcgacggcacccaggaggccgtcctcagctcctacgagtacgtcagccagggactcaagaagtacgacttcgacaacaccatgggaggcttctacatcgctcctgctttcatggacaagcttgttgtccatctctccaagaacttcatgaccctgcccaacatcaagatcccactcatcttgggtatctggggaggcaagggtcaaggaaaatccttccagtgtgagcttgtcttcgccaagatgggcatcaacccaatcatgatgagtgccggagagctggagagtggcaacgccggagagccagccaagctcatcaggcagcggtaccgtgaggctgcagacatgatcaagaagggtaagatgtgctgcctcttcatcaacgatcttgacgccggtgcgggtcggatgggcgggaccacacagtacaccgtcaacaaccagatggtgaacgccaccctcatgaacatcgccgatgcccccaccaacgtgcagctcccaggcatgtacaacaaggaggagaaccctcgtgtgcccatcgtcgtcactggtaacgatttctcgacgttgtacgcccctctgatccgtgatggtcgtatggagagggtatcttccagaccgaacaatgtcagcgacgagtccgtcgtcaagatcgtcgacaccttcccaggacaatccatcgactttttcggtgctctgcgtgctcgggtgtacgacgacgagtcgagaagctgcctagagtacgggcacatgctggtccaggagcaggacaatgtcaagcgtgtgcagcttgctgacacctacatgagccaggcagctctgggtgatgctaaccaggatgcgatgaagactggttccttctacggaggaggtaaaggggcacagcaaggtactttgcctgtgccggcaggatgcaccgaccagactgccaagaacttcgacccaacggcgaggagtgacgacggcagctgcctttacaccttttaa | 1257 |
| >transcript_19  atgagctccaaggccaacaggttcacagtcatggcagcggaaaacatcgacgagaagaggaacacggacaagtggaagggtcttgcgtacgatatctccgatgaccagcaggacatcaccagagggaagggcatcgtggactccctcttccaggcgcccacgggcgacggcacccaggaggccgtcctcagctcctacgagtacgtcagccagggactcaagaagtacgacttcgacaacaccatgggaggcttctacatcgctcctgctttcatggacaagcttgttgtccatctctccaagaacttcatgaccctgcccaacatcaagatcccactcatcttgggtatctggggaggcaagggtcaaggaaaatccttccagtgtgagcttgtcttcgccaagatgggcatcaacccaatcatgatgagtgccggagagctggagagtggcaacgccggagagccagccaagctcatcaggcagcggtaccgtgaggctgcagacatgatcaagaagggtaagatgtgctgcctcttcatcaacgatcttgacgccggtgcgggtcggatgggcgggaccacacagtacaccgtcaacaaccagatggtgaacgccaccctcatgaacatcgccgatgcccccaccaacgtgcagctcccaggcatgtacaacaaggaggagaaccctcgtgtgcccatcgtcgtcactggtaacgatttctcgacgttgtacgcccctctgatccgtgatggtcgtatggagagggtatcttccagaccgaacaatgtcagcgacgagtccgtcgtcaagatcgtcgacaccttcccaggacaatccatcgactttttcggtgctctgcgtgctcgggtgtacgacgacgagtcgagaagctgcctagagtacgggcacatgctggtccaggagcaggacaatgtcaagcgtgtgcagcttgctgacacctacatgagccaggcagctctgggtgatgctaaccaggatgcgatgaagactggttccttctacggaggaggtaaaggggcacagcaaggtactttgcctgtgccggcaggatgcaccgaccagactgccaagaacttcgacccaacggcgaggagtgacgacggagctgcctttacaccttttaatcatgccaatttaatatctgatgtttatcctttgtacctaagtaccgcatcgcatccaagcgattgctgggctatcccttgtacctaa | 1236 |
| >transcript_22  atgagctccaaggccaacaggttcacagtcatggcagcggaaaacatcgacgagaagaggaacacggacaagtggaagggtcttgcgtacgatatctccgatgaccagcaggacatcaccagagggaagggcatcgtggactccctcttccaggcgcccacgggcgacggcacccaggaggccgtcctcagctcctacgagtacgtcagccagggactcaagaagtacgacttcgacaacaccatgggaggcttctacatcgctcctgctttcatggacaagcttgttgtccatctctccaagaacttcatgaccctgcccaacatcaagatcccactcatcttgggtatctggggaggcaagggtcaaggaaaatccttccagtgtgagcttgtcttcgccaagatgggcatcaacccaatcatgatgagtgccggagagctggagagtggcaacgccggagagccagccaagctcatcaggcagcggtaccgtgaggctgcagacatgatcaagaagggtaagatgtgctgcctcttcatcaacgatcttgacgccggtgcgggtcggatgggcgggaccacacagtacaccgtcaacaaccagatggtgaacgccaccctcatgaacatcgccgatgcccccaccaacgtgcagctcccaggcatgtacaacaaggaggagaaccctcgtgtgcccatcgtcgtcactggtaacgatttctcgacgttgtacgcccctctgatccgtgatggtcgtatggagagggtatcttccagaccgaacaatgtcagcgacgagtccgtcgtcaagatcgtcgacaccttcccaggacaatccatcgactttttcggtgctctgcgtgctcgggtgtacgacgacgagtcgagaagctgcctagagtacgggcacatgctggtccaggagcaggacaatgtcaagcgtgtgcagcttgctgacacctacatgagccaggcagctctgggtgatgctaaccaggatgcgatgaagactggttccttctacggaggaggtaaaggggcacagcaaggtactttgcctgtgccggcaggatgcaccgaccagactgccaagaacttcgacccaacggcgaggagtgacgacggagctgcctttacaccttttaatcatgccaatttaatatctgatgtttatcctttgtacctaagtaccgcatcgcatccaagcgattgctgggctatcccttgtacctaa | 1236 |
